# Genetic Insights into Circulating Complement Proteins in Myalgic Encephalomyelitis/Chronic Fatigue Syndrome: A Potential Inflammatory Subgroup

**DOI:** 10.3390/ijms27031574

**Published:** 2026-02-05

**Authors:** Jessica Maya, Elizabeth R. Unger, Jin-Mann S. Lin, Mangalathu S. Rajeevan

**Affiliations:** Division of High-Consequence Pathogens & Pathology, Centers for Disease Control & Prevention, Atlanta, GA 30329, USAdwe3@cdc.gov (J.-M.S.L.)

**Keywords:** Myalgic Encephalomyelitis/Chronic Fatigue Syndrome (ME/CFS), complement system, genetics (pQTLs), heterogeneity, subgroups (genotype-stratified analysis)

## Abstract

Myalgic Encephalomyelitis/Chronic Fatigue Syndrome (ME/CFS) is a debilitating multi-system illness with heterogeneity that complicates identifying the pathophysiology, biomarkers, and therapeutic targets. Evidence indicates the importance of immune dysregulation, including the complement system, in ME/CFS. This study investigates the contribution of genetic drivers to potential dysregulation of the complement pathway in ME/CFS. We used protein quantitative trait loci (pQTL) analyses, adjusted for covariates using linear and logistic regression, to identify genetic variants significantly associated with plasma complement protein levels in a study sample identified from the general population (50 ME/CFS and 121 non-fatigued). ME/CFS patients carrying certain pQTLs exhibited dysregulation of the alternative complement pathway, which defined an inflammatory subgroup with a high C3/low Bb profile and established a genetic link to dysregulation of the alternative complement pathway. Six of the significant pQTLs were also associated with fatigue-related phenotypes in the UK Biobank, four of which were complement-associated, providing some validation in an independent population. Our findings highlight a mechanism by which risk alleles contribute to ME/CFS heterogeneity, providing evidence of a genetic basis for complement dysregulation in a subset of patients. This approach could identify pathway-focused subgroups in ME/CFS and related illnesses to inform personalized approaches to diagnosis and treatment.

## 1. Introduction

Myalgic Encephalomyelitis/Chronic Fatigue Syndrome (ME/CFS) is a debilitating multi-system illness characterized by substantial functional impairment accompanied by profound fatigue, post-exertional malaise, unrefreshing sleep, cognitive dysfunction, orthostatic intolerance, and a wide litany of additional symptoms that significantly reduce quality of life [1]. Its etiology remains elusive, with substantial heterogeneity complicating efforts to identify reliable biomarkers and therapeutic targets [2]; however, understanding the molecular basis of genotype/phenotype associations can greatly enhance our ability to tailor diagnostic and treatment strategies. While this approach is considered useful, it has not yet been investigated for many complex and heterogeneous diseases, including ME/CFS. Recently, we and others have tested whole-genome or pathway-focused genetic markers for their association with ME/CFS [3,4,5,6,7,8,9,10,11,12,13,14]. Many results from these earlier genetic association studies have not been replicated, possibly due to small sample sizes, inconsistent case ascertainment, genetic heterogeneity, disease time course, comorbid illnesses, and their combined interactions with environment, diet, medications, and microbial environments.

With the advent of high-throughput proteomic and genome-wide association studies, there has been a growing interest in linking disease-associated genetic variation to downstream molecular traits, including gene expression and circulating protein levels. These relationships are commonly investigated through quantitative trait loci analyses of expression (eQTL) and protein (pQTL), identifying genetic variants associated with changes in RNA or protein abundance, respectively [15]. These intermediate molecular phenotypes provide a mechanistic bridge between genotype and clinical presentation and offer a powerful framework for understanding complex disease biology and informing biomarker and therapeutic target discovery. While pQTL studies have been applied to cardiovascular diseases, inflammation-related diseases like multiple sclerosis, rheumatoid arthritis, ulcerative colitis, a variety of autoimmune disorders, and proteins in the human liver [15,16,17,18], their application to ME/CFS remains limited. Integrating genetic and proteomic data could improve understanding of disease susceptibility, progression, heterogeneity, and downstream immune dysregulation in ME/CFS.

Our group previously identified several genetic variants within the complement pathway that were associated with ME/CFS using a focused analysis of immune and inflammation-related genes [3]. The complement system is a critical component of innate immunity, consisting of plasma proteins that defend against infection and mediate inflammation through three activation pathways—classical, alternative, and lectin—all converging at C3 cleavage and tightly regulated to prevent chronic inflammation and immune-mediated damage [19,20]. Notably, our prior study identified two non-synonymous single-nucleotide polymorphisms (SNPs) in complement-related genes associated with ME/CFS: rs4151667 in *Complement Factor B* (*CFB*) and rs1061170 in *Complement Factor H* (*CFH*), both of which regulate the alternative pathway of complement activation. Of note, rs4151667 (L9H) and rs9337239 (E328D, also non-synonymous) are in high linkage disequilibrium (LD), reflecting non-random allele distribution and co-occurrence, and were found to be associated with ME/CFS by both allele and haplotype analysis [3]. These two SNPs are located in C2 and CFB, respectively, which are considered paralogous genes because of their evolutionary relationship through a gene duplication event and close proximity within the MHC Class III region of chromosome 6p21.3 [21,22,23]. Similarly, rs1061170 in *CFH* causes a tyrosine-to-histidine substitution at position 402 (Y402H) and is in high LD with three other SNPs in *CFH* (rs1061147, rs7529589, and rs1080155. Likewise, the major alleles of all four SNPs in *CFH* were associated with ME/CFS, further implicating the complement pathway in disease susceptibility. Interestingly, previous studies have reported complement dysregulation in ME/CFS, including elevated C4a responses, unique correlations between C3 levels and other circulating inflammatory proteins, and altered lectin and classical pathway components in plasma, cerebrospinal fluid, and extracellular vesicles [24,25,26,27,28,29,30,31].

It should be noted that *CFH* and *CFB* play opposing roles in the alternative complement pathway, with *CFB* promoting and *CFH* inhibiting the decay of C3 convertase [32]. Based on this observation, we hypothesize that components of the alternative pathway, including precursor proteins (C3 and Factor B), their breakdown products (C3a, C5a, and Bb), the negative regulator factor (Factor H), and the terminal complement activation marker (SC5b-9), are associated with ME/CFS, and that this association may be driven by the observed genetic variation in the complement proteins. Interestingly, ME/CFS-associated missense variants in these genes are also linked to age-related macular degeneration (AMD) but with inverted risk alleles [33,34]. As these alleles are associated with reduced complement activation and protection in AMD [35,36,37], we hypothesize that reduced complement activity contributes to disease risk in ME/CFS. These risk alleles could enable the identification of a subgroup of ME/CFS with complement dysregulation.

To extend our prior work, we investigate the association of plasma concentrations of complement proteins and related activation products (CRP, C3, Bb, Factor B, C3a, C5a, Factor H, and SC5b-9) with ME/CFS by identifying pQTLs among the 9146 SNPs that passed Affymetrix Human Immune and Inflammation Chip quality control [3]. We also identify correlations of complement proteins with measures of function, fatigue, and symptoms. Finally, we integrate these findings with previously reported genetic variants associated with ME/CFS illness to identify a genetic subgroup of ME/CFS with complement dysregulation. In addition, we sought to validate our identified complement- and disease-associated pQTLs with the UK Biobank’s publicly available datasets for post-viral and fatigue-related phenotypes. This integrative approach has the potential to identify biologically distinct subgroups within the heterogeneous ME/CFS population and should be helpful in studies of other chronic complex illnesses.

## 2. Results

### 2.1. Complement System Dynamics and Associations with Demographics and Disease Status

**Figure 1:**
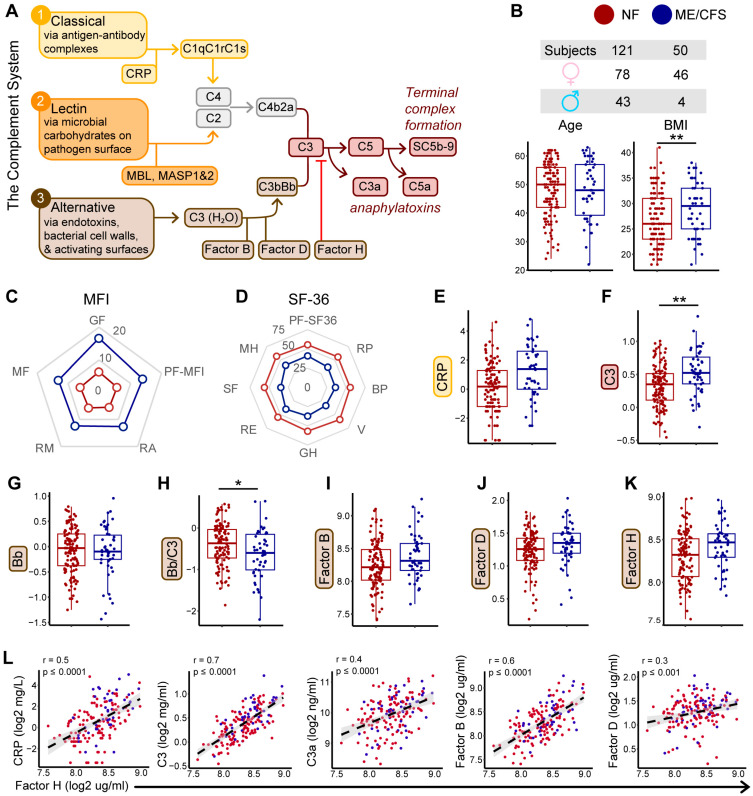
The complement system and associated component levels in ME/CFS compared with non-fatigued (NF) control subjects. (**A**) Schematic of the complement system and associated components, depicting the three activation pathways (color-coded: yellow—(1) classical; orange—(2) lectin; brown—(3) alternative; and maroon—C3 and subsequent products). The same color coding is used in other figures to facilitate linkage back to the complement pathway. (**B**) Demographic comparisons of sex, age (*p* = 0.96), and BMI (*p* = 0.007) between NF (red) and ME/CFS (blue) subjects (*n* = 171 total; 50 ME/CFS, 121 NF). Statistical comparisons for age and BMI were performed using a Wilcoxon rank-sum test. (**C**) Radar plot showing mean scores on Multidimensional Fatigue Inventory (MFI-20) subscales between NF and ME/CFS subjects, where lower MFI-20 scores indicate better health scores. GF = general fatigue; PF-MFI = physical fatigue; RA = reduced activity; RM = reduced motivation; MF = mental fatigue. (**D**) Radar plot showing mean T-scores on Short Form 36 survey (SF-36) subscales between NF and ME/CFS subjects, where a T-score of 50 refers to the norm of the US general population and lower SF-36 T scores signify a worse functional score: PF-SF36 = physical functioning; RP = role: physical; BP = bodily pain; V = vitality; GH = general health; RE = role: emotional; SF = social functioning; MH = mental health. (**E**–**K**) Log2-transformed plasma levels of complement proteins between ME/CFS and NF subjects after adjustment for covariates (Table 1): (**E**) C-Reactive protein (CRP, mg/L; *p_lin_* = 0.15, *p_log_* = 0.14), (**F**) C3 (mg/mL; *p_lin_* = 0.002, *p_log_* = 0.002), (**G**) Bb (mg/mL; *p_lin_* = 0.78, *p_log_* = 0.77), (**H**) Bb/C3 (*p_lin_* = 0.038, *p_lo__g_* = 0.038), (**I**) Factor B (µg/mL; *p_lin_* = 0.71, *p_log_* = 0.69), (**J**) Factor D (µg/mL; *p_lin_* = 0.16, *p_log_* = 0.14), and (**K**) Factor H (µg/mL; *p_lin_* = 0.07, *p_log_* = 0.07). Boxplots display the five-number summary: minimum (smallest value in the dataset), first quartile (Q1, 25th percentile), median (Q2, second quartile), third quartile (Q3, 75th percentile), and maximum (largest value). The central rectangle spans from the first quartile to the third quartile (the interquartile range (IQR)), a segment inside the rectangle shows the median, the diamond shows the mean, the vertical lines (sometimes referred to as whiskers 1.5xIQR) are extended to the extrema of the distribution in the data set, and the outliers are values outside the whisker range. Statistical comparisons of circulating complement protein levels were performed using a covariate-adjusted (Table 1) linear (*p_lin_*) and logistic (*p_log_*) regression analyses. * *p* ≤ 0.05, ** *p* ≤ 0.01. (**L**) Scatterplots depicting the extent of correlation (Pearson) of log2-transformed Factor H with (left to right): CRP (*p* = 6.1 × 10^−12^), C3 (*p* = 9.9 × 10^−28^), C3a (*p* = 4.3 × 10^−9^), Factor B (*p* = 1.4 × 10^−20^), and Factor D (*p* = 6.9 × 10^−4^) in all subjects (NF = red, ME/CFS = blue). Linear regression lines with 95% confidence intervals are overlaid.

With the complement system dynamics illustrated in Figure 1A, we next asked how these components vary with demographics and disease status. This study included 50 ME/CFS and 121 non-fatigued (NF) control Caucasian participants. Both groups were comparable in age but had significant differences in sex and body mass index (BMI) (Figure 1B). Among participants with ME/CFS, the median time since onset of fatigue was 8.97 years (range: 0.39–40.2 years), with 82.2% reporting a gradual illness onset [3]. Compared to the NF subjects, ME/CFS subjects had worse fatigue (higher scores) in all 5 domains of Multidimensional Fatigue Inventory (MFI-20, Figure 1C), greater symptom burdens in CDC symptom inventory (CDC-SI), and worse functional impairments (lower scores) in all 8 subscales of Short Form Health Survey (SF-36v2, Figure 1D).

Associations of the plasma concentrations of eight complement-related analytes among core proteins and activation products of the alternative pathway—CRP, C3, Bb, C3a, C5a, Factor B, Factor D, Factor H, and SC5b-9 with demographic covariates (age, sex, and BMI) in the overall study sample are shown in Table 1 and Figure S1A–K. The purpose of these analyses was to identify demographic factors that systematically influence complement protein levels across the study population in order to inform covariate adjustment in subsequent case–control and subgroup analyses. BMI was the most consistent covariate, showing moderate yet significant positive associations with seven of the nine measured proteins: CRP, C3, C3a, C5a, Factor B, Factor D, and Factor H (all *p* < 0.0001), strongest (R^2^) with C3 (Table 1, Figure S1A–G). In contrast, Bb and SC5b-9 were not significantly associated with BMI. The Bb/C3 ratio is commonly used as an index of relative alternative pathway activity because Bb reflects pathway activation while C3 represents overall complement abundance; in this analysis, however, the inverse association with BMI was likely driven by increased C3 levels rather than reduced Bb abundance or altered alternative pathway activation (Table 1, Figure S1H). Sex was significantly associated with CRP and Factor B (Table 1, Figure S1I,J), with lower levels observed in males. Only Factor D levels were associated with age (Table 1, Figure S1K). The demographic factors listed in Table 1 were therefore included as covariates in downstream analyses of the associated proteins.

To evaluate illness-associated differences in complement protein levels, we performed both linear and logistic regression analyses comparing protein concentrations between illness groups while adjusting for covariates. Linear regression assessed differences in protein concentrations as continuous outcomes, while logistic regression modeled the odds of ME/CFS as a function of protein levels. Box plots of log2-transformed complement protein levels stratified by disease status are shown in Figure 1E–K and Figure S1L–N, while Table 2 summarizes ME/CFS case–control associations after adjustment for the demographic covariates identified in Table 1. Although several proteins exhibited small shifts in median levels between ME/CFS and NF, these differences were modest in magnitude, and only C3 and the Bb/C3 ratio were significantly associated with ME/CFS across both models. Specifically, the linear model indicates that C3 levels differ significantly between the two groups, while the logistic model shows that higher C3 levels are associated with increased odds of ME/CFS.

We examined correlations among all measured complement proteins across the full study population to assess coordinated regulation within the complement pathway and to provide biological context for downstream genetic analyses (Table S1). Factor H associations (Figure 1L) demonstrated the most extensive inter-protein correlation profile across the complement panel, including moderate relationships with CRP, C3, C3a, Factor B, and Factor D. The correlation of Factor H was much stronger with C3 (r = 0.7. *p* ≤ 0.0001) and Factor B (r = 0.6. *p* ≤ 0.0001) and showed no significant correlation with Bb (r = 0.11, *p* = 0.12). Given that the alternative complement cascade consists of multiple interdependent and tightly regulated components (Figure 1A), the majority of the pairwise comparisons showed significant positive associations (Table S1).

### 2.2. Associations Between Complement System Components and Functional Health Scores

**Figure 2:**
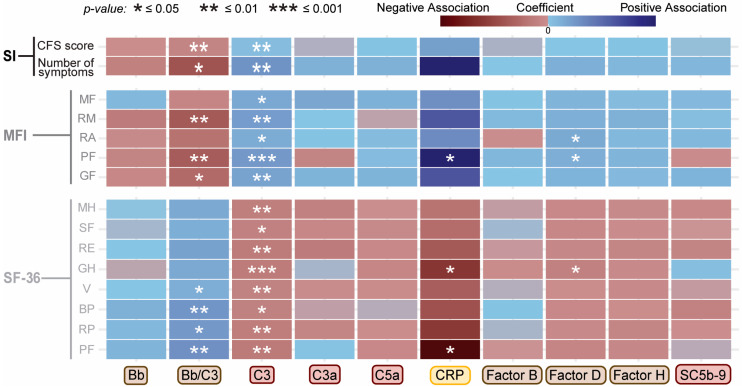
Association of circulating complement protein levels with participants’ function and symptom scores. Heatmap depicting covariate-adjusted associations between plasma complement protein levels and scores from three questionnaires: Symptom Inventory (CDC-SI, top), Multidimensional Fatigue Inventory (MFI-20, middle), and Short Form 36 survey (SF-36 T scores, bottom). Lower SF-36 T scores indicate worse functional scores, whereas lower MFI-20 and CDC-SI scores indicate better health scores. Rows represent individual domains or subscales for each instrument, and columns represent complement-related proteins. Color represents the coefficient, or magnitude, of each association, with blue indicating a positive association, while red/maroon indicates a negative association. Statistical comparisons were performed using covariate-adjusted linear regression analysis. * *p* ≤ 0.05, ** *p* ≤ 0.01, *** *p* ≤ 0.001. (SI score = CDC Symptom Inventory; MFI: MF = mental fatigue, RM = reduced motivation, RA = reduced activity, PF = physical fatigue, GF = general fatigue; SF-36: MH = mental health, SF = social functioning, RE = role: emotional, GH = general health, V = vitality, BP = bodily pain, RP = role: physical, PF = physical functioning). NOTE: Complement protein labels are color-coded to match components of the complement system illustrated in Figure 1A.

Results of covariate-adjusted linear regression modeling between each analyte and the measures of function (SF-36), fatigue (MFI-20), and symptoms (CDC-SI) are shown as a heatmap in Figure 2 and as correlation plots in Figure S2A–D. C3 shows the most consistent pattern among the tested complement proteins, with higher plasma levels associated with greater symptom burden, increased fatigue, and lower functioning scores (Figure S2B). CRP exhibited the largest beta coefficients across several MFI-20 and SF-36 domains, indicating that small increases in severity or functional impairment were associated with relatively large shifts in CRP levels; however, the corresponding R^2^ values remained low (Figure S2C; R^2^ ~ 0.32), suggesting that despite strong directional effects, the symptom scores explained only a modest proportion of the overall variance in participants’ CRP levels. Factor D and the Bb-to-C3 ratio also showed significant associations with some MFI and SF-36 metrics. In contrast, Bb, C3a, C5a, Factor B, Factor H, and SC5b-9 showed no significant associations with any individual scores. While these analyses were performed in the total study population, stratified analyses by disease status did not reveal meaningful differences, indicating that the pooled results adequately represent associations in both ME/CFS cases and controls.

### 2.3. Identification of Genetic Variants Impacting Plasma Levels of Complement Proteins

**Figure 3:**
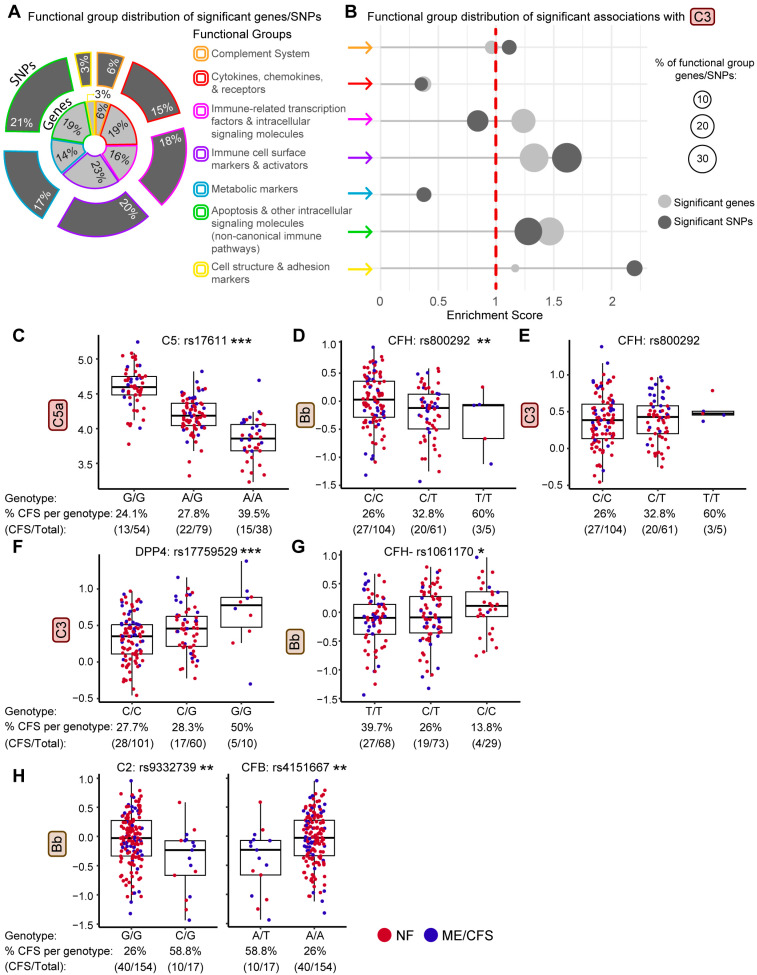
Functional group distribution of complement protein-associated SNPs/genes and complement protein levels by genotype and illness status. (**A**) Donut plot summarizing the distribution across 7 functional groups of single-nucleotide polymorphisms (SNPs—dark gray outer ring) and corresponding genes (light gray inner ring) associated with complement proteins (*p* ≤ 0.01 after covariate-adjustment). Functional group assignments were based on annotated gene functions for genes and associated SNPs (Tables S2–S12). (**B**) Lollipop plot showing functional group enrichment among SNPs and genes significantly associated with C3 plasma levels, following the pQTL analysis between complement protein levels and SNPs (776 SNPs (359 genes) were identified as significantly associated with at least 1 complement protein). Dot size reflects the proportion of significant functional group genes (light gray) and SNPs (dark gray). Enrichment score > 1 (dotted red line) indicates overrepresentation. (**C**–**H**) Boxplots of plasma protein levels (log2, mg/mL) by genotype. Dot color indicates disease status (NF = red; ME/CFS = blue); *x*-axis = percentage of ME/CFS subjects within each genotype group; (**C**) C5a levels for rs17611/C5 genotypes (*p* = 1.61 × 10^−27^, log2, ng/mL), a pQTL that has been validated in other cohorts, serving as an internal control in our dataset; (**D**) Bb for rs800292/*CFH* genotypes (*p* = 0.003); (**E**) C3 for rs800292/*CFH* genotypes (*p* = 0.61); (**F**) C3 for rs17759529/*DPP4* genotypes (*p* = 0.0002); (**G**) Bb for rs1061170/*CFH* genotypes (*p* = 0.025); (**H**) left: Bb for rs4151667/*CFB* genotypes (*p* = 0.002), right: Bb for rs9332739/*C2* genotypes (*p* = 0.002). The percentage of ME/CFS subjects within each genotype group is indicated on the *x*-axis, while the (CFS/Total) row indicates the number of ME/CFS subjects vs. the total number of subjects per genotype. Boxplots represent the median ± 25th and 75th quartiles. Whiskers represent 1.5× the interquartile ranges. Outliers are values outside the whisker range. Asterisks indicate a significant overall genotype effect based on a covariate-adjusted full-versus-reduced (FvR) linear regression model comparing models with and without the genotype term. Significance (*p*-value) reflects improvement in model fit attributable to genotype and does not represent pairwise comparisons between individual genotype groups. * *p* ≤ 0.05, ** *p* ≤ 0.01, *** *p* ≤ 0.001.

To investigate if genetic variants are associated with circulating complement protein levels and to identify biological pathways enriched or depleted among these associations, we performed a quantitative trait locus (pQTL) analysis using all 9146 SNPs previously analyzed in this population [3], identifying 3192 SNPs significantly associated with at least one complement protein (*p* < 0.05). Data on the SNPs associated with each protein are presented in the supplement (Tables S2–S11). To distill meaningful biologic themes from this large data set, the 776 SNPs with *p* < 0.01, representing 359 genes, were categorized into one of seven curated functional groups [(1) direct association with the complement system (20 genes), (2) cytokines, chemokines, and their receptors (69 genes), (3) immune-associated transcription factors and intracellular immunomodulators (57 genes), (4) immune cell surface markers and activators (82 genes), (5) metabolic markers (50 genes), (6) apoptosis and other intracellular signaling molecules involved in non-canonical immune pathways (70 genes), and (7) cell structure and adhesion markers (11 genes); Table S12]. The distribution of the 776 significant SNPs and 359 genes by functional group is shown graphically in Figure 3A.

This framework enabled analysis of functional group enrichment scores for each complement protein association (C3 in Figure 3B, and all other proteins in Figure S3A–I). For C3, we observed overrepresentation in the immune cell surface markers and activators, apoptosis, and cell structure and adhesion functional groups, whereas SNPs and genes related to cytokine and chemokine receptors and metabolic markers were underrepresented. By organizing significant pQTLs by protein and functional group categories, this approach facilitates prioritization and biological interpretation of key genetic associations that would be difficult to discern when considering the full set of 776 significant SNP associations. Statistical parameters for each protein’s most significant associations are found in Supplemental Tables S2–S11. For example, the rs17611 SNP in *C5* emerged as one of the top-ranked pQTLs for C5a in this population (Figure 3C) in agreement with previous reports in independent cohorts [38,39], and serving as an internal control. Two SNPs, rs800292 in *CFH* and rs17759529 in *DPP4*, significantly associated with Bb and C3 levels, are also among the most significant in this pQTL screen (Figure 3D–F). Genotypes of rs800292/*CFH,* previously implicated in complement regulation, showed a progressive decrease in Bb levels across genotype categories under an additive model, with lower Bb levels observed with increasing copies of the T allele (Figure 3D). Interestingly, rs800292 shows a similar decrease in Bb/C3 ratios (Figure S4A), with a slight inverse trend seen in genotype-stratified C3 levels (Figure 3E). The G allele of rs17759529 in *DPP4*, a top-ranked pQTL for CRP and C3 in the over-represented immune cell surface markers functional group, was associated with higher C3 levels in homozygous carriers (Figure 3F). Notably, DPP4 is a multifunctional protease expressed on immune cells and endothelium that has been implicated in SARS-CoV-2 through proposed roles in viral entry and immune regulation [40]. Additionally, the major allele T in rs1061170 (*CFH*) was associated with lower Bb levels (Figure 3G). Bb levels were similarly significantly lower in individuals carrying the minor C allele at rs9332739 (*C2*) and the T allele at rs4151667 (*CFB*), two SNPs in linkage disequilibrium (LD) (Figure 3H). The box plots in Figure 3D–H also show the skewed distribution of ME/CFS cases by genotype of these complement pQTLs, suggesting an interaction between complement pQTLs and ME/CFS risk.

### 2.4. Identification of Genetic Variants Impacting Both Plasma Levels of Complement Proteins and Disease Status

We identified 16 SNPs found in both the 3192 protein-associated SNPs and the 48 previously reported ME/CFS-associated SNPs [3], 11 of which act in the complement cascade (Table 3). The additional immune and inflammatory pathways represented (T cell signaling, Chemokine, G-protein coupled receptor signaling, and TNF superfamily signaling) indicate genetic risk for ME/CFS beyond the complement system.

Among the 16 SNP associations, four were predicted to act in cis (defined as variants located within 1Mb of the transcription start site of the impacted protein’s associated gene), such as variants located in *C2*, *CFB*, or *CFH* impacting levels of Bb, Factor B, or Factor H, respectively (Table 3). The remaining 12 variants were predicted to be trans-pQTLs, acting elsewhere in the genome (e.g., *SERPINA5* impacting C3, *GRK4* impacting C3, and *PDE4D* impacting C3a and C5a). Of note, rs3020729 (*CD8A*, miRNA binding) was significantly associated with higher levels of CRP and C3 levels, and rs2277680 (*CXCL16*, missense) impacted Factor H and Factor B levels (Table 3). By contrast, rs9550987 (*TNFRSF19,* missense) was associated with decreased levels of C3 and Factor H (Table 3).

### 2.5. Directionality of pQTL Effects on Complement Proteins and ME/CFS Risk

To further characterize how disease-associated genetic variants influence complement protein dynamics, we examined the directionality of pQTL effects on circulating complement levels in relation to ME/CFS risk. Specifically, we examined the strength and direction of the impact of the genetic variants (pQTLs) on both the disease and the molecular trait, providing genetic insight into the functional/causative associations between the disease and its deep phenotypes.

**Figure 4:**
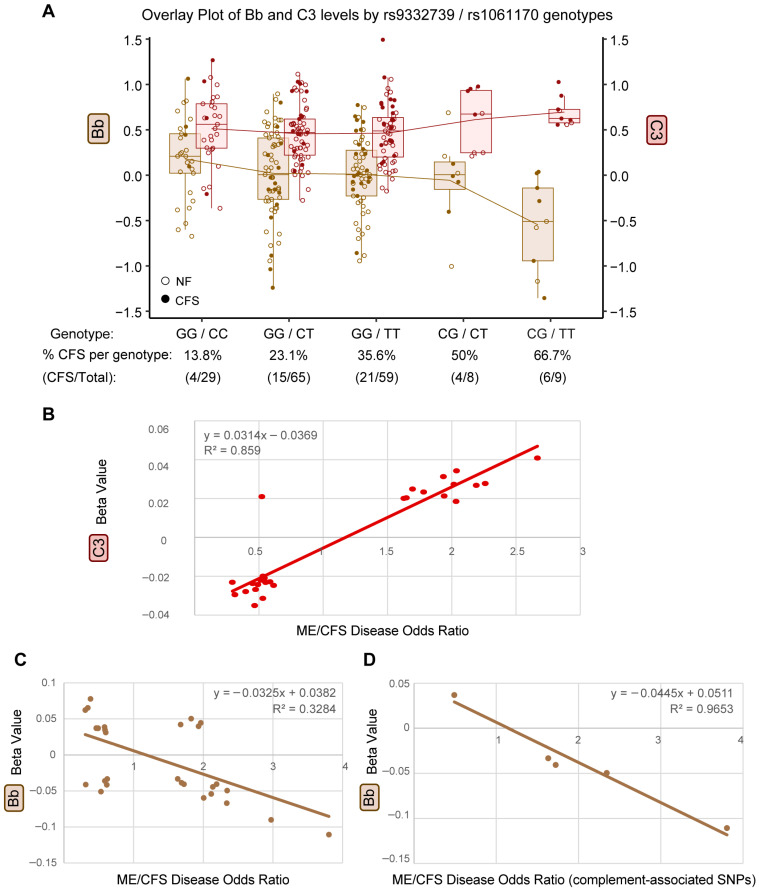
Directionality of pQTL impact on both disease (odds ratio (OR) for ME/CFS) and molecular trait. (**A**) Overlay boxplots of Bb (brown, primary *y*-axis, log2, mg/mL) and C3 (maroon, secondary *y*-axis, log2, mg/mL) plasma levels by genotype combinations of rs9332739/C2 and rs1061170/CFH (NF = empty dot, ME/CFS = filled dot). The percentage of ME/CFS subjects within each genotype group is indicated on the *x*-axis, while the (CFS/Total) row indicates the number of ME/CFS subjects vs. the total number of subjects per genotype. Boxplots represent the median ± 25th and 75th quartiles. Whiskers represent 1.5× the interquartile ranges. Outliers are values outside the whisker range. The red or orange line links the mean plasma levels of C3 and Bb, respectively, across the five genotype combinations. (**B**,**C**) Linear regression plot showing the relationship between odds ratios (OR) for disease risk and beta coefficients (β) for plasma protein levels, with each point representing one SNP. All associations are done with respecte to the minor allele frequency in cases and controls: (**B**) C3 (27 SNPs included, *X*-axis—OR, *Y*-axis—β); (**C**) Bb (30 SNPs included, *X*-axis—OR, *Y*-axis—β); (**D**) Bb (6 SNPs associated with compliment pathway included, *X*-axis—OR, *Y*-axis—β). NOTE: Complement protein labels and data points/lines are color-coded to match components of the complement system illustrated in Figure 1A.

Genotype-stratified analyses revealed reciprocal trends between C3 levels and Bb-related measures across multiple disease-associated variants. For example, inverse relationships between genotype-stratified C3 levels and Bb/C3 ratios were observed for rs800292 (*CFH*) (Figure 3E and Figure S4A) and similarly for the rs9332739 (*C2*) risk allele (Figure 3H and Figure S4B,C). When rs9332739 (*C2*) and rs1061170 (*CFH*) were considered jointly, five genotype combinations ordered by increasing number of ME/CFS-associated risk alleles showed opposing trends in Bb and C3 levels (Figure 4A). The double-risk genotype (CG–TT) exhibited the lowest Bb levels, the highest C3 levels, and the highest proportion of ME/CFS cases, indicating a graded relationship between combined genetic burden, complement protein imbalance, and disease prevalence. Together, these patterns point to a genetically determined imbalance between complement precursor (C3) and activation product (Bb).

To evaluate whether this imbalance aligns with disease risk across a broader set of variants, we correlated the strength of genetic association with ME/CFS (odds ratios, OR) with the magnitude of pQTL effects (β coefficients) for C3 and Bb (Figure 4B–D). This analysis included 57 SNPs overlapping between disease-associated variants and pQTLs for C3 (27 SNPs) and Bb (30 SNPs), as summarized in Table S13, with all associations evaluated with respect to the minor allele. A strong positive correlation was observed between disease OR and C3 β values (R^2^ = 0.859; Figure 4B), indicating that increasing genetic risk for ME/CFS is associated with higher C3 levels. In contrast, disease OR showed an inverse relationship with Bb β values (R^2^ = 0.328; Figure 4C). Notably, when analysis was restricted to complement-pathway SNPs, disease OR and Bb β values exhibited a near-perfect correlation (R^2^ = 0.965; Figure 4D), reinforcing a model in which genetic risk for ME/CFS is associated with elevated upstream complement activity, skewed toward increased C3 and reduced Bb.

### 2.6. Use of Genotype-Defined Complement pQTLs to Identify ME/CFS Subgroups

We initially assessed a non-genetic model with complement proteins and fragments for their performance as predictive markers, using ROC analysis where the outcome was ME/CFS status. In this analysis, CRP, Factor B, and C3 had the highest predictive power, with Area Under the Curve (AUCs) of 0.75, 0.75, and 0.69, respectively (Figure S5A). Only C3 was statistically significant in this non-genetic model.

To assess whether incorporating pQTLs into the model could identify biologically distinct subgroups with improved discrimination of ME/CFS status, we repeated ROC analyses as above, but with subjects stratified by genotype-defined groups based on pQTLs significantly associated with complement proteins or disease status (Table 4), further restricted to those within the complement system functional group (Table S12). Results were filtered for AUC > 0.75, *p* < 0.05, and sufficient group size (n ≥ 15) and are summarized in Table 4. Heterozygotes for two SNPs, rs9332739 in *C2* (genotype CG) and rs800292 in *CFH* (genotype CT), previously examined in Figure 3, showed a high AUC (0.84 ± 0.05; *p*-value = 0.0004; Table 4) with a total of 71 subjects (26 ME/CFS and 45 NF) for further exploratory analysis.

**Figure 5:**
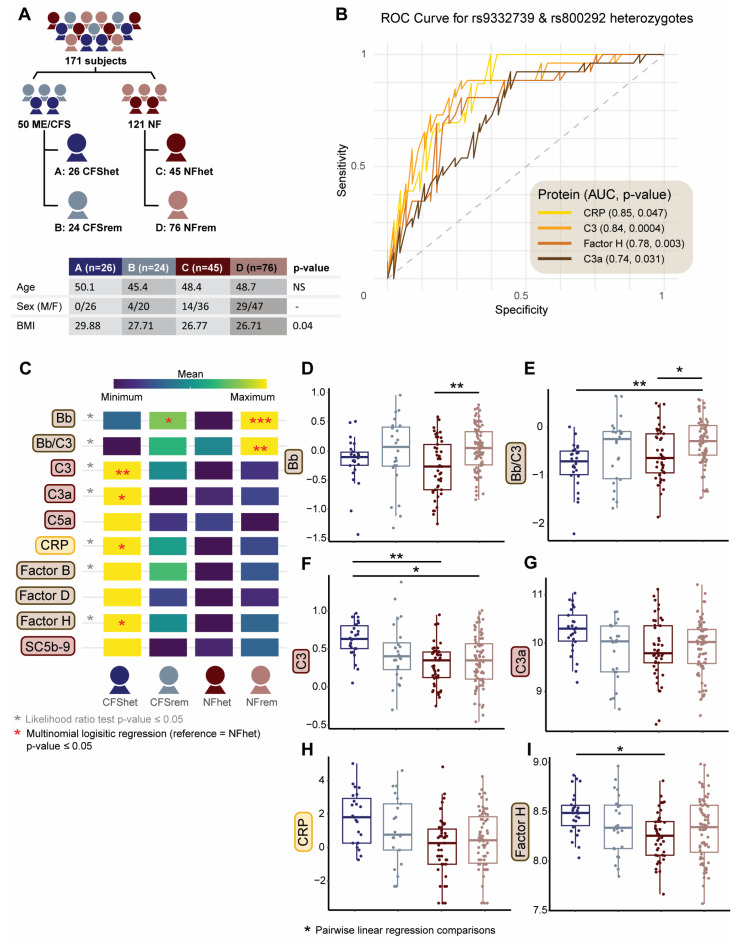
Stratification of ME/CFS and non-fatigued control subjects using complement protein-associated pQTL genotypes. (**A**) Subgroup distribution and demographics (age, sex, BMI) based on genotype status of rs9332739 (*C2*) and rs800292 (*CFH*). Group A (CFShet, dark blue) = ME/CFS heterozygous at one or both loci; Group B (CFSrem, light blue) = Remainder of ME/CFS subjects; Group C (NFhet, dark red) = NF controls heterozygous at one or both loci; Group D (NFrem, pink) = remainder of NF controls. (**B**) Complement protein ROC curves for ME/CFS (yellow = CRP, light orange = C3, dark orange = Factor H, brown = C3a), restricted to heterozygous groups (Group A (CFS het) and Group C (NFhet). (**C**) Heatmap based on the log2-transformed mean range for each complement protein, low (blue) to high (yellow). Mean values for genotype-defined subgroups (CFShet, CFSrem, NFhet, NFrem) are shown. Asterisks indicate statistical significance, adjusted for covariates (gray = likelihood ratio test with *p* ≤ 0.05, red = subgroup-specific multinomial logistic regression with reference = NFhet and *p* ≤ 0.05). (**D**–**I**) Boxplots showing distributions for plasma levels of complement protein (log2, mg/mL) by genotype group, CFShet = dark blue, CFSrem = light blue, NFhet = dark red, NFrem = pink. (**D**) Bb (NFhet vs. NFrem *p* = 0.002), (**E**) Bb/C3 (CFShet vs. NFrem *p* = 0.003; NFhet vs. NFrem *p* = 0.03), (**F**) C3 (CFShet vs. NFhet *p* = 0.005, CFShet vs. NFrem *p* = 0.02), (**G**) C3a, (**H**) CRP, and (**I**) Factor H (CFShet vs. NFhet *p* = 0.05). Boxplots display the five-number summary: minimum (smallest value in the dataset), first quartile (Q1, 25th percentile), median (Q2, second quartile), third quartile (Q3, 75th percentile), and maximum (largest value). The central rectangle spans from the first quartile to the third quartile (the interquartile range (IQR)), a segment inside the rectangle shows the median, the diamond shows the mean, the vertical lines (sometimes referred to as whiskers 1.5xIQR) are extended to the extrema of the distribution in the data set, and the outliers are values outside the whisker range. Statistical comparisons of circulating complement protein levels (black asterisk) were performed using a covariate-adjusted (Table 1) pairwise linear regression analysis. * *p* ≤ 0.05, ** *p* ≤ 0.01, *** *p* ≤ 0.001.

To further evaluate whether complement-associated pQTLs could be used to define biologically meaningful subgroups and to compare the relative discriminatory performance of complement protein markers within those genotype-defined strata, all 171 subjects were stratified into four groups based on heterozygosity at rs9332739 in *C2* and rs800292 in *CFH*. ME/CFS participants with heterozygous genotypes at one or both SNPs were assigned to CFShet (n = 26, Group A), while the remaining ME/CFS cases were designated as CFSrem (n = 24, Group B). Non-fatigued controls were similarly divided into NFhets (n = 45, Group C) and NFrem (n = 76, Group D), with the distribution and subgroup demographics shown in Figure 5A. Because subgrouping was defined by heterozygosity, two individuals with the double risk genotype for rs800292 but no risk allele at rs9332739 were assigned to the CFSrem and NFrem groups. The CFShet group contained no male participants, and BMI was significantly different between the CFShet group and the other three groups. Within this genotype-stratified framework, CRP and C3 exhibited improved AUCs in the heterozygous-defined CFShets vs. NFhets comparison, with AUC values of 0.85 and 0.84, respectively (Table 4, Figure 5B), while Factor H and C3a also had enhanced predictive power (0.78 and 0.74 AUC, respectively) over the non-stratified model (Figure S5A). These exploratory analyses compare relative discriminatory performance across genotype-defined subgroups and illustrate an approach that could be applied in larger populations to inform biologically meaningful subgrouping in ME/CFS.

To assess complement protein differences across genotype-stratified illness groups, we applied three statistical approaches: a likelihood ratio test to evaluate overall statistical model fit, a multinomial logistic regression model to identify proteins associated with subgroup assignment (using NFhet as the reference), and pairwise covariate-adjusted linear regression models to examine direct groupwise differences. The likelihood ratio test identified seven proteins—Bb, Bb/C3, C3, C3a, CRP, Factor B, and Factor H—that significantly improved the statistical model, indicating that these proteins contribute meaningfully to group classification (Figure 5C). Multinomial regression revealed that C3, C3a, CRP, and Factor H levels were significantly associated with increased odds of belonging to the CFShet group relative to NFhet (Figure 5C), suggesting potential utility as subgroup classifiers. Pairwise linear regression comparisons confirmed several between-group differences (Figure 5D–I), including lower Bb and Bb/C3 in NFhet compared to NFrem, and lower Bb/C3 in CFShet compared to NFrem. The CFShet group also demonstrated higher C3 and Factor H levels relative to NFhet, with CFShet C3 levels significantly higher than NFrem as well. Collectively, these complementary analyses demonstrate statistically robust associations between circulating complement proteins and genotype-defined subgroups—even after adjusting for covariates—highlighting their potential role in distinguishing biologically meaningful ME/CFS subgroups.

**Figure 6:**
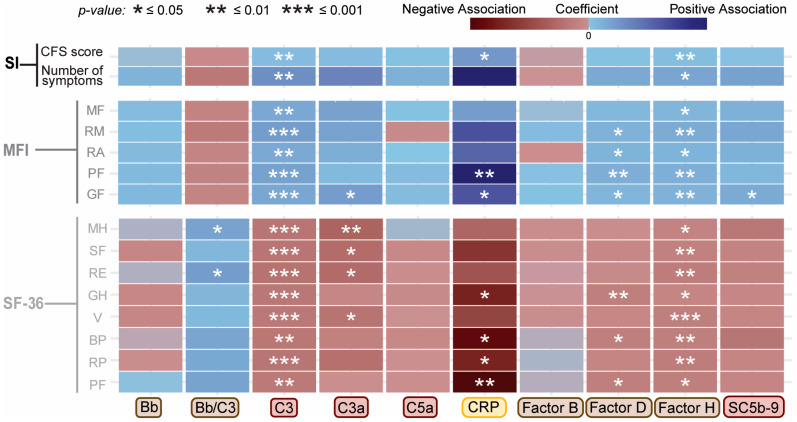
Associations between complement protein levels and symptom/function scores for genotype-restricted subgroup (CFShet, NFhet, n = 71). Heatmap of covariate-adjusted associations between complement protein levels and scores: Symptom Inventory (SI, top), Multidimensional Fatigue Inventory (MFI, middle), and Short Form 36 survey (SF-36 T scores, bottom). Rows represent individual domains or subscales for each survey, and columns represent complement-related proteins. Color represents the coefficient, or magnitude, of each association, with blue indicating a positive association and red indicating a negative association. Statistical comparisons were performed using covariate-adjusted linear regression analysis. * *p* ≤ 0.05, ** *p* ≤ 0.01, *** *p* ≤ 0.001. (SI = CDC Symptom Inventory; MFI: MF = mental fatigue; RM = reduced motivation; RA = reduced activity; PF = physical fatigue; GF = general fatigue; SF-36: MH = mental health; SF = social functioning; RE = role: emotional; GH = general health; V = vitality; BP = bodily pain; RP = role: physical; PF = physical functioning). NOTE: Complement protein labels are color-coded to match components of the complement system illustrated in Figure 1A.

We examined the covariate-adjusted association between complement protein levels and symptom and functional scores in the heterozygous subgroups (CFShet and NFhet; Figure 6 and Figure S5B–D). The covariate-adjusted regression models in this reduced population (n = 71) revealed multiple significant associations between complement protein levels and scores in CDC-SI, MFI-20, and SF-36. In the total study sample population (Figure 2), only four complement-related measurements (Bb/C3, C3, CRP, and Factor D) showed significant associations, whereas seven complement proteins showed significant associations across at least one of the three survey domains in the genotype-restricted groups (Figure 6). These included newly significant relationships for C3a, Factor H, and SC5b-9 (Figure 6 and Figure S5B–D). For the total and genotype-restricted samples, the correlations followed the same direction. While these subgroup analyses are exploratory due to sample size and subgroup composition, this expanded set of associations suggests that complement dysregulation may be more tightly linked to reported functional impairments in genetically defined subgroups, reinforcing the relevance of host genotypes in shaping disease heterogeneity in ME/CFS. Further evaluation of this approach in larger, independent cohorts, including cross-cohort comparisons, will be necessary to assess its full utility.

### 2.7. Comparison of Significant SNPs Between Our Population and Fatigue-Related Phenotypes in the UK Biobank

To evaluate the consistency and broader applicability of a pQTL-based approach for identifying genetic contributors to ME/CFS, we compared SNPs associated with each circulating complement protein (Tables S2–S11, top 50 SNPs from each table with the lowest *p*-values) identified in our population to SNPs associated with fatigue traits in the publicly available UK Biobank GWAS data for Chronic Fatigue Syndrome, Post-Viral Fatigue Syndrome, a broader fatigue and malaise population labeled by the UK Biobank as 41202/R53, and Ever CFS, a group that the UK Biobank defined as individuals reporting recovery from ME/CFS [41,42]. Six SNPs across five genes overlapped between our pQTL dataset and the UK Biobank fatigue-related traits (Table 5), mapping to complement-related genes *CFB, CFH*, and *C2*, as well as *MMP10* (involved in the breakdown of extracellular matrix) and *PIK3R5* (which plays roles in cell growth, proliferation, differentiation, motility, survival, and intracellular trafficking). Four of the six SNPS (three genes) were among those we identified as both pQTLs and ME/CFS-associated (rs641153, rs800292, rs9332739, and rs4151667). Interestingly, rs394811 in *PIK3R5* was the only SNP to overlap with the UK Biobank’s designated CFS group. In contrast, SNPs in *CFB*, *CFH*, and *C2* were associated with the broader fatigue and malaise and post-viral fatigue phenotypes. Moreover, the direction of effect for each overlapping SNP—including risk allele identity and odds ratio—was consistent between the UK Biobank phenotypes and our population, providing some replication of the biological relevance of these shared associations.

## 3. Discussion

This study developed a novel pQTL-based approach to address clinical heterogeneity in ME/CFS using genotype and pathway-focused stratification. Our analysis focused on complement pQTLs and yielded several noteworthy findings. First, we observed significantly higher levels of C3 in ME/CFS compared to NF controls, alongside a decrease in the ratio of Bb to C3, suggesting altered alternative pathway activation in this disease. Second, we identified multiple complement pQTLs that were also associated with disease status. These pQTLs included polymorphisms in *C2*/*CFB* (rs9332739 and rs4151667 in LD) and *CFH* (rs800292). The ability of complement protein levels to distinguish between ME/CFS and NF controls improved when the analysis was limited to the genotype-restricted subgroup (CFShet vs. NFhet). Cases and controls in this subgroup not only differed in C3 and Factor H levels but also showed distinct patterns across multiple analyses that improved the model’s ability to distinguish between the four subgroups and improved the odds of subgroup assignment, with proteins in the genotype-restricted group also exhibiting more extensive associations with functional impairment and patient health scores. Additionally, several pQTLs that we identified in our population with functional consequences (non-synonymous SNPs: rs4151667, rs9332739, rs641153, rs800292, rs394811, and rs17293607) were found to overlap with SNPs that were significantly associated with fatigue-related phenotypes listed in the UK Biobank, demonstrating how integrating approaches may help resolve inconsistencies across genetic studies of this heterogeneous disease. These findings stress a link between complement system genetics, complement protein dysregulation, and ME/CFS pathophysiology, while also providing an approach for the much-needed subgrouping of heterogeneous diseases such as ME/CFS.

Our association results reinforce the need to adjust data for covariates, such as age, sex, and BMI, when evaluating differences in complement components between ME/CFS and healthy controls; adjustments are likely important for other protein associations as well. Our findings of the influence of age, sex, and BMI on the level of these proteins are largely in agreement with recently published results [43,44,45], requiring them to be used as covariates in the analysis of complement proteins using genetic or non-genetic models. The complement dysregulation identified in our study of ME/CFS persisted after covariate adjustment, highlighting its disease-specific nature rather than a demographic artifact. While age is known to influence certain classical complement components, such as declining C1q levels in older adults [46,47], we only included age as a covariate in models where it was empirically significant for our specific population, such as for Factor D. In contrast, BMI emerged as a consistent covariate across multiple complement proteins—most notably C3—likely reflecting adipose-driven complement protein production [48,49,50,51]. This is particularly relevant for ME/CFS, where symptom severity and metabolic shifts may influence body composition. Of note, earlier studies that did not control for BMI reported no differences in C3 between ME/CFS and controls [24,52]. Sex was also considered when statistically warranted (Factor B and CRP), and adjustment ensured that complement differences were not driven by the unbalanced sex ratio observed in ME/CFS [53]. Sex hormones and X-linked immune genes shape distinct immunologic profiles [54], and women generally exhibit lower baseline complement levels [55,56]. Notably, the CFShet subgroup contained no male participants, suggesting potential sex-dependent genotype effects that warrant future stratified analyses, in line with other recent suggestions of ME/CFS immune subtyping by sex [31,57].

C3 is the central protein in the complement cascade, regulated by three major pathways (classical, lectin, and alternate pathways) which lead to a pathway-dependent C3 convertase converting C3 to C3b and C3a. The classical/lectin pathways generate C3 convertase by combining C4bC2b, whereas the alternative pathway contributes to C3 convertase (C3bBb) by binding of C3b to Bb (Bb results from cleavage of Factor B by Factor D). An increase in plasma Bb is considered a marker of complement activation, whereas a decrease in Bb indicates complement inhibition resulting from the inhibitory functions of Factor H [19]. High C3 and C3a, along with high CRP (previously reported in other ME/CFS populations [58,59]) hint at ongoing innate immune activation; however, concurrent high Factor H (an alternative pathway inhibitor) and low Bb suggest increased complement inhibition in our ME/CFS subjects. This could reflect a compensatory response or genetically driven dampening of the alternative pathway in ME/CFS, especially in variant-carrying subgroups. Of note, a recent plasma proteomic analysis in ME/CFS suggests a similar hypothesis of attenuated complement activity, reflecting a compensatory mechanism and subsequent exhausted immune state in ME/CFS [60]. Further measurements and functional assays are necessary to fully understand the role of the alternative pathway in this disease, as well as the contributions of the classical and lectin pathways to the high levels of C3 that we see associated with ME/CFS. Integrating genetic variant data, we found subjects carrying the risk allele in *C2/CFB* and *CFH* (rs9332739 and rs1061170) had lower levels of Bb in plasma, and that Bb levels could be impacted by the pQTL rs800292 in *CFH*, although there was only a trend associating the latter trans-pQTL with illness (Armitage *p* = 0.09). Notwithstanding, heterozygotes for rs9332739 in *C2* and rs800292 in *CFH* resulted in the highest AUC for prediction of illness status. Both rs9332739 (E318D) in *C2* and rs800292 (V62I) in *CFH* are missense pQTLs for Bb, and both are reported to be protective for AMD [32,33,34,35,36,37,61].

There is limited information on the functional role of these pQTLs in the alternate pathway. Other studies have documented their association with diseases that have seemingly unrelated clinical presentations and pathologies. For example, studies of AMD and atypical hemolytic uremic syndrome (aHUS), clinically different illnesses but both having overactivity of the alternative pathway, identified defective regulation of the alternative pathway [62,63]. This differs from our finding that at least a subset of ME/CFS subjects show inhibition of the alternative pathway through what appears to be a genetically determined low level of Bb. Moreover, when *C2* and *CFH* variants were considered together, Bb and C3 levels showed stepwise opposing trends across the five genotype combinations, most stark in the genotype group with the highest ME/CFS proportion. These reciprocal trends point to a genetically encoded imbalance between the complement precursor (C3) and the activation product (Bb), potentially reflecting dysregulated convertase activity in ME/CFS. Correlation analyses between C3 or Bb protein levels and disease odds ratios revealed consistent directional concordance across diverse genetic associations, reinforcing a model in which ME/CFS genetic risk is linked to elevated upstream complement activity skewed toward C3 and away from Bb.

Higher complement inhibition, reflected by reduced Bb levels observed in our study, is consistent with prior reports showing that the minor allele of rs800292 in *CFH* inhibits the alternative pathway through stronger binding of the variant to C3b [64,65]. Factor H and C3 protein were highly correlated and present in high levels in the ME/CFS group, which may reflect increased inhibition of the alternate pathway by Factor H [66]. Understanding these biological relationships is essential for interpreting circulating levels of complement proteins in ME/CFS, as disease-associated changes may reflect both direct dysregulation and broader shifts in pathway dynamics. For example, rs800292 in *CFH* is reported as a potent regulator of matrix metalloproteinase-8 (MMP-8), a proinflammatory enzyme conferring risk for cardiovascular diseases, and carriers of the rs800292 minor allele show reduced serum MMP-8 levels [67]. CFH resides within the regulators of complement activation (RCA) gene cluster on chromosome 1, a region known to harbor variants that can modulate complement activity in both activating and inhibitory directions across disease contexts [65,68]. Notably, we identified one cis-acting pQTL for Factor H, negatively regulating rs1065489 in *CFH*. This study also identified four disease-associated pQTLs for C3 (rs6108 in *SERPINA5,* rs3020729 in *CD8A*, rs1801058 in *GRK4,* rs9550987 *in TNFRSF19*). Although C3 levels were significantly different between illness groups, we did not identify any co-localized cis-QTL impacting both illness status and C3 level; however, circulating C3 levels were decreased by the minor alleles of three cis acting intronic pQTLs (rs7257062, rs2241393, rs2241392), all of which performed as strong predictors of illness status, with AUC > 0.78 and *p*-values below 0.006. Subjects homozygous for the major alleles of these three variants had higher C3 levels but, for unclear reasons, were not associated with ME/CFS. Finally, several pQTLs within the *TRAF1/C5* region on chromosome 9 influenced circulating C5a levels but were not associated with ME/CFS status. One such variant, rs7037673, has been reported as a pQTL for C5 expression in prefrontal cortex tissue [69], highlighting potential tissue-specific effects and supporting further investigation into complement-related neuroimmune pathways in ME/CFS. Taken together, these observations illustrate how complement-associated pQTLs can provide biologically informative markers for prioritization and hypothesis generation; however, broader mechanistic interpretation requires consideration of sample size, population context, and replication across independent cohorts.

Dysregulated complement activity in ME/CFS mirrors persistent complement activation, impaired metabolic and immune responses, and altered inflammatory cytokine profiles, consistent with chronic inflammation and incomplete post-infection resolution illnesses [70,71,72,73,74,75,76,77,78,79] reported in other infection-associated chronic conditions and illnesses (IACCIs). Understanding the nuanced differences in complement profiles across IACCIs may help explain why some individuals recover while others develop chronic sequelae. Related but mechanistically distinct complement system patterns are observed in other chronic immune or neurological conditions: in lupus, low CRP with C3/C4 consumption impairs nuclear antigen clearance, whereas in ME/CFS, studies have found elevated CRP noted its elevation alongside high Factor H and reduced Bb, suggesting suppressed clearance and unresolved inflammation driven by host genetics [80,81,82]. In multiple sclerosis (MS), complement activation contributes to demyelination, with high Factor H observed in some patients, despite a clear complement locus yet to be identified– consistent with predominantly environmental/secondary involvement [83,84]. In Alzheimer’s disease, complement-associated variants and insufficient control of complement-mediated synaptic pruning implicate complement in neurodegeneration [85], which may be relevant to the cognitive impairments seen in many ME/CFS patients despite differences between the two conditions. Notably, our identification of a trans-pQTL for Factor H in *TNFRSF19*—a gene expressed in the central nervous system—suggests a link between peripheral complement regulation and neuroimmune signaling in ME/CFS. Ultimately, these findings position complement dysregulation not as an isolated feature but as a potential hallmark of chronic immunoinflammatory disease, offering a framework for therapeutic targeting and for refining biological subgroups across heterogeneous conditions.

In summary, our study demonstrates a pQTL-based approach integrating genotype with protein biomarker data that can uncover immune subtypes within a heterogeneous disease, offering a path forward for more personalized treatment strategies and mechanistic investigation. In addition, our study identifies inflammation driven by high C3—likely resulting from increased inhibition of the alternative pathway—as a central feature in a genetically defined subgroup of ME/CFS. This subgroup, identifiable by complement pQTLs, may benefit from targeted therapies such as C3-lowering agents [86]. The complement abnormalities we observe mirror those observed in other IACCIs, in which imbalanced complement activity—whether genetically inherited or acquired—contributes to chronic inflammation and tissue damage. Our findings contribute to this landscape by highlighting genetic variants in the complement system as heritable contributors to ME/CFS susceptibility, where low basal alternative pathway activity may predispose individuals to persistent inflammation following an infectious or environmental insult. Complement also intersects with metabolic, endothelial, and coagulation pathways, linking dysregulation to hallmark ME/CFS symptoms like fatigue and bioenergetic stress [87].

The strengths of this study include the use of participants from the population classified using a standardized approach and employing a novel pQTL analysis. Limitations include the relatively small number of ME/CFS patients, lack of racial diversity, and cross-sectional data. Further study is required to evaluate the generalizability of our findings. Future studies should expand this approach genome- and proteome-wide and leverage resources such as the DecodeME or SearchMECFS biorepository to explore stratification strategies across diverse populations. In parallel, tracking complement dynamics longitudinally—especially in patients with high-risk genotypes or post-infection—could clarify disease trajectories and inform therapeutic targeting.

## 4. Materials and Methods

### 4.1. Subject Recruitment and Characteristics

This study included the 50 ME/CFS and 121 non-fatigued (NF) control subjects from the previously published genetic analysis focusing on inflammatory and immune-related pathways [3]. Briefly, participants were identified in the follow-up phase of the longitudinal study of ME/CFS in Georgia. ME/CFS cases were identified with the operationalized 1994 international research case definition [88]. Sample characteristics such as age, sex, body mass index (BMI), illness duration and onset, and symptom and functioning scores across fatigue (multidimensional fatigue inventory, MFI-20), functional status (SF-36), CDC symptom inventory (CDC-SI), and routine clinical tests results (including hs-CRP) were recorded for every participant [3]. The source study was approved by the Institutional Review Board of the Centers for Disease Control and Prevention and Abt Associates (now Abt Global). All subjects provided written informed consent for participating in the study and for anonymous testing and storage of biological samples.

### 4.2. Plasma Protein and Complement Component Assays

Whole blood from each subject was collected in ethylenediaminetetraacetic acid (EDTA) tubes and processed to obtain plasma within 30 min to minimize ex vivo activation of complements. Plasma was separated, aliquoted, and stored at −70 °C. A frozen plasma aliquot for each subject was shipped to the CLIA-certified Complement Laboratory at National Jewish Health, Denver, Colorado, for analysis of complement proteins/activation products using their validated clinical assays. Basic assay methods included measuring levels of C3a, C5a, Bb, SC5b-9, and Factor D by ELISA, C3 by nephelometry, and Factor B and Factor H by radial immunodiffusion. Results of hs-CRP testing were retrieved from the study database.

### 4.3. Statistical Analyses

#### 4.3.1. Data Transformation

Statistical and genetic analyses were performed on log2-transformed values of the measured proteins involved in the complement system (C3a, C5a, C3, Bb, SC5b-9, Factor D, Factor B, Factor H, and CRP). This data transformation was based on the observed variance and distribution of protein levels across all participants and was performed using RStudio (2025.05.1+513).

#### 4.3.2. Data Analyses and Visualizations for Group Phenotypes and Complement Protein Levels

A Wilcoxon rank-sum test was used to test for significant differences in age and BMI between ME/CFS and NF control subjects. Radar plots created in Microsoft Excel were used to display the mean values for MFI-20 and SF-36 T-scores for both groups. A Pearson correlation test was performed to assess the association between complement factor levels.

#### 4.3.3. Identification of Confounding Factors

A linear regression analysis was used to determine the bivariate association of circulating complement protein levels with demographic features (or variables) recognized to be associated with ME/CFS, such as age, sex, and BMI. Covariate adjustments specific to each circulating complement protein based on these associations and those supported by the literature were included in downstream analyses.

#### 4.3.4. Analysis of Circulating Complement Proteins Between ME/CFS and NF Subjects

Either binary or multinomial logistic regression analyses were performed to test whether individual complement proteins were significantly associated with group classification, maintaining covariate considerations. In the 2-group analysis, ME/CFS cases status (Y = 1) vs. *Total NF* controls (Y = 0) was modeled as the binary outcome:

$$logit\left(P(Subject=Total\;NF)\right)=\;\beta_{0}+\;\beta_{1}\left({log}_{2}(Protein\;levels)\right)+\;\beta_{2}\left(Covariate\right)\ldots +\;\varepsilon$$

In the 4-group multinomial model, subgroup assignment (Y = one of the four groups) was used as the categorical outcome, with *NFhet* set as the reference category:

$$log\frac{P\left(Group=k\right)}{P\left(Group=NFhet\right)}=\;\beta_{0}+\;\beta_{1}\left({log}_{2}(Protein\;levels)\right)+\;\beta_{2}\left(Covariate\right)\ldots +\;\varepsilon$$

For k ∈ {CFShet, CFSrem, NFrem}

Likelihood ratio tests (LRTs) were performed to assess the overall contribution of each complement protein to the model. For each protein, we compared the full model (including the protein of interest as a predictor) against a reduced model (excluding the protein) using the following:

$$LRT\;statistic\;=\;-2\;x\;\left(log\;likelihood\;of\;reduced\;model-log\;likelihood\;of\;full\;model\right)$$

where the full model is: $Group\;\sim \;{log}_{2}(Protein\;levels)+Covariates$

And the null model is: Group ~ Covariates

Pairwise linear regression analyses were performed to directly compare log2-transformed *Protein levels* between groups while adjusting for covariates. These were applied to both the 2-group and 4-group comparisons:

$${log}_{2}(Protein\;levels)\;=\;\beta_{0}+\;\beta_{1}\left(Group\right)+\;\beta_{2}\left(Covariate\right)\ldots +\;\varepsilon$$

Group was treated as a binary or categorical variable, and pairwise *p*-values were extracted using estimated marginal means and post hoc contrasts to highlight specific group differences. Boxplots and dot graphs were created using the R package ggPlot2 (R version 2025.05.1+513) to visualize protein distributions across groups.

#### 4.3.5. Analysis of Participant Survey Data with Circulating Complement Proteins

Linear regression modeling with previously noted covariate adjustments was performed to assess complement protein levels with participant survey data. Specifically, log2-transformed plasma *Protein levels* were modeled as a function of continuous survey metrics (CDC-SI, MFI-20, and SF-36), using the formula:

$${log}_{2}\left(Protein\;level\right)=\;\beta_{0}+\;\beta_{1}\left(functional/health\;score\right)+\;\beta_{2}\left(Covariate\right)\ldots +\;\varepsilon$$

RStudio was used to create a heatmap to display the regression β_1_ coefficient and the *p*-values for each association. To illustrate specific trends, representative correlation dot plots with corresponding R squared values were created for analytes with significant associations, capturing the distribution of case- and control-level survey responses.

#### 4.3.6. Analysis of SNP Associations with Circulating Complement Proteins

A genotypic logistic regression using an additive model was previously applied to assess 9146 SNPs that passed Affymetrix Human Immune and Inflammation Chip quality control and their associations with ME/CFS [3]. To investigate genetic variants for associations with complement components, we performed covariate-adjusted linear regression analyses in RStudio comparing full and reduced models for each SNP-protein pair. This analysis was conducted across all subjects, without stratification by illness group, to assess genetic regulation of complement protein expression. For each SNP-protein pair, a full linear model including the SNP genotype and relevant covariates was compared with a reduced model excluding the genotype.

Full model:

$${log}_{2}\left(Protein\;level\right)=\;\beta_{0}+\;\beta_{1}\left(SNP\;genotype\;coded\;additively\right)+\;\beta_{2}\left(Covariate\right)\ldots +\;\varepsilon$$

Reduced model:

$${log}_{2}\left(Protein\;level\right)=\;\beta_{0}+\;\beta_{2}\left(Covariate\right)\ldots +\;\varepsilon$$

An F-statistic was calculated to compare the full and reduced models for each SNP–protein pair, testing whether the inclusion of genotype significantly improved model fit. The *p*-value from the full-versus-reduced (FvR) comparison was used to define significance. Additionally, a Bonferroni correction was applied to account for multiple testing across all models, and these results are reported in Supplementary Tables S2–S11. Allele frequencies, including identification of major and minor alleles for the genetic analyses, were determined using data from this study population.

#### 4.3.7. Pathway Enrichment Analysis to Annotate SNP Associations with Circulating Complement Protein Levels

The linear regression analysis identified 3192 SNPs significantly associated with circulating complement proteins (full vs. reduced linear regression model, *p* < 0.05). The 3192 SNPs associated with circulating complement protein levels were further filtered to identify top associations (full vs. reduced linear regression model, *p* < 0.01) for each measured complement protein. This analysis identified 776 significant SNPs linked to complement protein plasma levels, representing a total of 359 unique genes. To investigate the distribution of significant SNPs across functional groups for each protein, data was extracted from Metascape, Golden Helix SVS software version 7.0, and the literature to create a list of all genes with SNPs significantly associated with each complement protein and their function, allowing assignment to one of seven functional groups (Table S12). For each complement protein, the number of significant genes and SNPs associated with each functional group was calculated. Enrichment scores were computed by dividing the observed proportion of genes/SNPs in a functional group within each complement factor’s “top hits” list by the expected proportion of genes/SNPs in each functional group based on the overall distribution of significant genes/SNPs across all functional groups and complement factor “top hits” lists. The overall distribution of significant genes/SNPs across all functional groups, regardless of complement factor association, was visualized by creating a donut plot using Microsoft Excel. Lollipop plots were generated using R to visualize enrichment scores for each circulating complement protein measured, with the *y*-axis containing functional groups; overrepresentation in a group indicated by an enrichment score > 1.

#### 4.3.8. Analysis of SNP Associations with Circulating Complement Protein Levels and Disease Status

The 3192 SNPs significantly associated with circulating complement proteins were compared against the previously published 32 functional SNPs, 10 proxy SNPs, and 6 additional pyrosequenced complement-linked SNPs significantly associated with ME/CFS (with sex and BMI covariate adjustments) [3], identifying 16 SNPs in common with both analyses and therefore associated with both disease status and circulating complement protein levels. These pQTLs were also assessed for whether they were cis- or trans-acting using the QTLbase home database (http://www.mulinlab.org/qtlbase (accessed on 13 November 2025). In these analyses, effect sizes are reported as β ± SE, where β represents the estimated per-allele change in log2-transformed protein abundance associated with genotype under an additive model, adjusted for covariates.

#### 4.3.9. Analysis of the Directionality of Genetic Variant Traits with Disease Risk

To investigate the broader pattern of disease-associated SNPs with complement protein levels, we assessed the relationship between the odds ratios (ORs) of significant genetic variants for ME/CFS risk and beta coefficients for circulating complement protein levels. ORs were derived from previously published SNP-disease associations (27 SNPs assessed against C3 beta values, and 30 SNPs assessed against Bb beta values, Table S13) [3], and beta coefficients were generated from our circulating complement protein-level QTL analyses using the log2-transformed plasma concentrations of each complement protein. Specifically, we plotted ORs on the *x*-axis and corresponding beta values for C3 or Bb on the *y*-axis, with each point representing a single SNP significantly associated with ME/CFS status.

#### 4.3.10. Genotype-Stratified Analysis to Identify ME/CFS Subgroup Related to Complement System Dysregulation

The pQTLs identified in this study were evaluated for their ability to discriminate between illness status and subject subgroups in the study. This was done by first prioritizing pQTLs with high power for the prediction of illness status by conducting a series of ROC analyses that split the subjects based on genotypes of QTLs, with log2-transformed plasma complement protein levels as the test variable. Specifically, participants were grouped by genotype at each SNP of interest, and logistic regression models were used to calculate predicted probabilities for ME/CFS status. These probabilities were then used to compute ROC curves and AUC values. Genotypes that resulted in better discrimination of subjects’ illness status, as indicated by a high Area Under the Curve (AUC ≥ 0.75), were selected for combined analysis of markers to further improve the AUC for predicting illness status. An example of grouping subjects based on two markers, rs9332739 and rs800292, was visualized to evaluate the discriminatory capacity of complement-associated pQTLs for potential subgroup identification within the study population. This combined marker analysis, conducted using rs9332739 and rs800292, classified ME/CFS participants with heterozygous genotypes at either SNP as Subgroup A (CFShet), and the remainder (those without heterozygosity at either locus) were assigned to Subgroup B (CFSrem). Similarly, NF controls with heterozygous genotypes at either marker were assigned to Subgroup C (NFhet), and all remaining controls were assigned to Subgroup D (NFrem). Of note, no individuals carried the homozygous risk genotype for rs9332739 (C/C), while five individuals carried the homozygous risk genotype for rs800292 (T//T). This four-group classification was applied to assess refined stratification of disease status and to characterize potential genotype-informed subgroups. Logistic models were rerun using these stratified groups to evaluate differences in complement protein levels, as described in Section 4.3.4, and ROC performance across identified subgroups.

#### 4.3.11. Comparison of Significant SNPs Between Our Population and the UK Biobank

To assess overlap between our top genetic variant associations and previously reported ME/CFS-related SNPs, we compared the top 50 SNP associations for each of the nine measured complement proteins with publicly available genome-wide association data from the UK Biobank [41,42]. Specifically, we extracted SNPs significantly associated with ME/CFS-related phenotypes defined by the UK Biobank, including the groups Chronic Fatigue Syndrome, Post-Viral Fatigue Syndrome, the ICD-10-coded “Fatigue and Malaise” category (UK Biobank field 41202; code R53), and Ever CFS, which denotes individuals who self-reported a prior diagnosis of ME/CFS, including those who reported recovery. These standardized phenotype definitions are established by the UK Biobank and are widely used in population-level genetic analyses. These phenotypes were selected for their relevance and comparability to ME/CFS-related symptom profiles, allowing assessment of overlap between complement protein-associated pQTLs identified in our population and genetic associations reported for fatigue-related traits in a large independent population. We evaluated whether any of the top complement protein-associated SNPs in our dataset overlapped with those reported for these UK Biobank phenotypes and compared variant annotations, *p*-values, and odds ratios for common SNPs.

## Figures and Tables

**Table 1:** Association of demographic factors with plasma complement proteins and activation products (log2 transformed) determined by linear regression analyses.

Plasma Component or Fragment *	Covariate	Coefficient	Std Error	Adjusted R^2^	F Statistic	*p*-Value
CRP	BMI	0.169	0.024	0.230	47.47	1.3 × 10^−10^
C3		0.033	0.004	0.269	63.11	2.7 × 10^−13^
C3a		0.036	0.008	0.102	20.28	1.2 × 10^−5^
C5a		0.025	0.005	0.106	21.06	8.7 × 10^−6^
Factor B		0.026	0.005	0.142	28.32	3.3 × 10^−7^
Factor D		0.020	0.004	0.113	22.62	4.2 × 10^−6^
Factor H		0.023	0.004	0.181	38.47	4.2 × 10^−9^
Bb/C3		−0.027	0.008	0.066	12.92	4.3 × 10^−4^
CRP	Sex	0.645	0.318	0.019	4.10	4.5 × 10^−2^
Factor B		0.159	0.061	0.034	6.84	9.8 × 10^−3^
Factor D	Age	0.011	0.002	0.117	23.44	2.9 × 10^−6^

* No association of Bb and Sc5b-9 with any of the demographic factors tested in this study. Complement protein labels are color-coded to match components of the complement system illustrated in Figure 1A.

**Table 2:** Association of illness group with plasma complement proteins and activation products (log2 transformed) determined by linear regression analyses.

Plasma Component or Fragment	Illness Group	Mean ± SD	β ± SE *	*p*-Value **
Bb (µg/mL)	NF	−0.077 ± 0.45	0.022 ± 0.078	0.779
	ME/CFS	−0.099 ± 0.51		
Bb/C3	NF	−0.404 ± 0.50	0.184 ± 0.088	0.038
	ME/CFS	−0.638 ± 0.59		
C3 (mg/mL)	NF	0.324 ± 0.31	−0.151 ± 0.047	0.0016
	ME/CFS	0.539 ± 0.32		
C3a (ng/mL)	NF	9.90 ± 0.58	−0.083 ± 0.094	0.378
	ME/CFS	10.06 ± 0.57		
C5a (ng/mL)	NF	4.218 ± 0.40	−0.044 ± 0.063	0.483
	ME/CFS	4.312 ± 0.36		
CRP (mg/L)	NF	0.318 ± 1.71	−0.424 ± 0.292	0.148
	ME/CFS	1.405 ± 1.83		
Factor B (µg/mL)	NF	8.242 ± 0.35	−0.021 ± 0.058	0.712
	ME/CFS	8.385 ± 0.35		
Factor D (µg/mL)	NF	1.233 ± 0.29	−0.066 ± 0.047	0.158
	ME/CFS	1.327 ± 0.32		
Factor H (µg/mL)	NF	8.299 ± 0.29	−0.081 ± 0.044	0.066
	ME/CFS	8.427 ± 0.26		
SC5b9 (ng/mL)	NF	7.113 ± 0.60	−0.038 ± 0.097	0.694
	ME/CFS	7.152 ± 0.51		

* Estimates (β) represent adjusted differences in mean log2-transformed protein levels computed as Total NF − Total ME/CFS. Negative values, therefore, indicate higher protein levels in ME/CFS cases. ** Relationship of plasma complement/activation products with ME/CFS adjusted for covariates: C3a, C5a, C3, and Factor H adjusted for BMI; Factor B was adjusted for BMI and sex; Factor D was adjusted for age and BMI. No covariate adjustment needed for Bb and SC5b9. Complement protein labels are color-coded to match components of the complement system illustrated in Figure 1A.

**Table 3:** Genetic variants impacting both disease status and plasma levels of measured complement proteins and activation factors (log2-transformed).

Pathway	Chromosome: Gene Name	SNP rsID	ME/CFS Risk Allele (nt)	Variant Consequence	Impacted Complement Protein (cis/trans)	β ± SE *	*p*-Value
Complement cascade	6: *C2*	rs9332739 ~	Minor (C)	Missense (E318D)	Bb/C3	−0.49 ± 0.13	1.9 × 10^−4^
					Bb (cis)	−0.36 ± 0.12	0.002
					Factor B (cis)	−0.23 ± 0.08	0.007
	6: *CFB*	rs4151667 ~	Minor (T)	Missense (L9H)	Bb/C3	−0.49 ± 0.13	1.9 × 10^−4^
					Bb (cis)	−0.36 ± 0.12	0.002
					Factor B (cis)	−0.23 ± 0.08	0.007
		rs641153	Major (C)	Missense (R32L)	Factor B (cis)	0.24 ± 0.07	0.001
					Bb/C3	−0.24 ± 0.10	0.021
					Factor D (trans)	−0.11 ± 0.05	0.046
	1: *CFH*	rs7529589 ^	Major (G)	Intronic	Factor H (cis)	0.06 ± 0.03	0.020
					Bb (trans)	0.10 ± 0.05	0.036
		rs1061147 ^	Major (G)	Codon-synonymous (A307A); splicing regulation	Bb (trans)	0.12 ± 0.05	0.015
		rs800292	Minor (T)	Missense (V62I)	Bb (trans)	−0.19 ± 0.06	0.003
					Bb/C3	−0.21 ± 0.07	0.003
		rs1061170 ^	Major (T)	Missense (H402Y)	Bb (trans)	0.11 ± 0.05	0.025
		rs10801555	Major (G)	Intronic	Bb (trans)	0.12 ± 0.05	0.014
	3: *MASP1*	rs3774268	Minor (T)	Missense (S445R)	Bb/C3	−0.24 ± 0.08	0.003
					Bb (trans)	−0.17 ± 0.07	0.026
	14: *SERPINA5*	rs6115	Minor (G)	Missense (S64N)	Bb/C3	−0.11 ± 0.05	0.047
		rs6108	Minor (A)	UTR-3; miRNA binding	C3 (trans)	0.07 ± 0.03	0.026
T cell signaling	2: *CD8A*	rs3020729	Major (T)	UTR-3; miRNA binding	CRP (trans)	−0.67 ± 0.25	0.008
					C3 (trans)	−0.10 ± 0.05	0.028
Chemokine	17: *CXCL16*	rs2277680	Major (G)	Missense (I142T; A200V)	Factor H (trans)	−0.06 ± 0.03	0.024
					Factor B (trans)	−0.08 ± 0.04	0.031
G-protein coupled receptor signaling	5: *PDE4D*	rs2014012	Major (A)	Intronic	C3a (trans)	−0.13 ± 0.06	0.036
					C5a (trans)	−0.09 ± 0.04	0.038
	4: *GRK4*	rs1801058	Major (C)	Missense (V486A); splicing regulation	C3 (trans)	−0.07 ± 0.03	0.034
TNF superfamily signaling	13: *TNFRSF19*	rs9550987	Minor (T)	Missense (S31T); splicing regulation	C3 (trans)	0.09 ± 0.03	0.011
					Factor H (trans)	0.07 ± 0.03	0.027

* β ± SE denotes the regression coefficient (β) and its standard error (SE) from covariate-adjusted linear pQTL models. β represents the estimated per-allele change in log2-transformed circulating protein levels associated with the indicated SNP (additive model), while SE reflects the statistical uncertainty of this estimate. SNP IDs underlined indicate pQTLs that have not been previously reported (no match in the QTL database, accessed on 3 February 2026: http://www.mulinlab.org/qtlbase). ~ and ^ indicate SNPs that are in linkage disequilibrium (LD).

**Table 4:** ROC analysis of complement protein markers stratified by genotype-defined pQTLs for identifying ME/CFS subgroups.

Variant	Gene	Genotype	Test Variable *	Covariates	# of CFS	# of NF	# of Subjects	AUC ± SE	95% CI	*p*-Value
Single marker analysis										
rs9332739	*C2/CFB*	CG	Factor H	BMI	10	7	17	0.89 ± 0.09	0.71–1	0.05
rs800292	*CFH*	CT	C3	BMI	20	41	61	0.82 ± 0.06	0.74–0.94	0.001
			Factor H					0.78 ± 0.06	0.7–0.94	0.007
			CRP	Sex, BMI	17	40	57	0.84 ± 0.05	0.72–1	0.04
rs1061170		TT	Factor B	Sex, BMI	27	40	67	0.81 ± 0.06	0.7–0.92	0.03
rs10801555		GG	Factor B	Sex, BMI	26	38	64	0.81 ± 0.06	0.7–0.92	0.03
rs395544		CC	Factor B	Sex, BMI	20	36	56	0.83 ± 0.05	0.73–0.94	0.03
rs1065489		GG	C3	BMI	27	61	88	0.75 ± 0.06	0.64–0.86	0.02
rs7135975	*C1R*	AG	C3	BMI	16	43	59	0.76 ± 0.08	0.6–0.92	0.01
rs7257062	*C3*	CC	C3	BMI	20	43	63	0.78 ± 0.06	0.65–0.91	0.006
rs2241393		CC	C3	BMI	18	38	56	0.81 ± 0.06	0.69–0.93	0.003
rs2241392		CC	C3	BMI	19	43	62	0.78 ± 0.07	0.65–0.91	0.006
rs7037673	*C5*	CT	C3	BMI	16	45	61	0.82 ± 0.05	0.72–0.93	0.005
			Bb/C3					0.81 ± 0.06	0.69–0.93	0.006
rs261753	*C9*	CC	CRP	Sex, BMI	31	76	107	0.78 ± 0.05	0.69–0.87	0.04
rs1986158	*CR1*	CT	C3	BMI	11	23	34	0.83 ± 0.09	0.65–1	0.009
2-marker analysis										
			CRP	Sex, BMI	23	43	66	0.85 ± 0.05	0.76–0.94	0.046
rs9332739 and rs800292	*C2/CFH*	CG/CT	C3	BMI	26	45	71	0.84 ± 0.05	0.74–0.94	0.0004
			Factor H					0.78 ± 0.06	0.67–0.89	0.003

* For single marker and 2-marker analyses, only test variables with AUC > 0.75 and *p* < 0.05 are listed. ROC analyses were used to evaluate the ability of log2-transformed complement protein levels to discriminate ME/CFS cases from non-fatigued controls within genotype-defined pQTL subgroups. AUC values summarize discriminatory performance for single-marker and two-marker genotype strata. Genotype: SNP genotype(s) used to define the subgroup for analysis. Test variable: Log2-transformed complement protein used in the ROC model. AUC ± SE: Area under the ROC curve with standard error, summarizing discriminatory performance within the genotype-defined subgroup. *p*-value: Significance of AUC relative to the null (AUC = 0.5). # = Number.

**Table 5:** Overlapping SNP associations between circulating complement proteins and UK Biobank fatigue-related phenotypes.

UK Biobank Fatigue-Related Phenotype	Chr: Gene Name	Variant	Variant Consequence (Reference nt— Variant nt)	UK Biobank: Cases/ Healthy Controls	SNP—UK Biobank Phenotype Odds Ratio	SNP—UK Biobank Phenotype Odds Ratio Confidence Interval	SNP—UK Biobank Phenotype *p*-Value	SNP— Impacted Complement Protein *p*-Value
Chronic Fatigue Syndrome	17: *PIK3R5*	rs394811	Synonymous (G-A)	2047/307,792	0.847	0.75–0.96	0.007	0.002 (C3)
Post-Viral Fatigue Syndrome	6: *CFB*	rs641153	Missense (G-A)	4363/216,118	0.571	0.38–0.86	0.005	0.001 (CFB)
	11: *MMP10*	rs17293607	Missense (C-T)	4360/216,027	0.920	0.86–0.98	0.009	0.004 (C5a)
41202/R53 Fatigue and Malaise	1: *CFH*	rs800292	Missense (G-A)	2132/283,735	0.754	0.61–0.94	0.010	0.003 (Bb)
Ever CFS	6: *C2*	rs9332739	Missense (G-C)	2547/121,864	1.186	1.05–1.34	0.008	0.002 (Bb)
	6: *CFB*	rs4151667	Missense (T-A)	2547/121,863	1.185	1.05–1.34	0.009	0.002 (Bb)

Chr = Chromosome number.

## Data Availability

Data supporting the reported results are provided in the Supplementary Materials section. Researchers who want to access the datasets used in this study should email the CDC’s ME/CFS Program (cfs@cdc.gov) and discuss next steps for the data request. The ME/CFS program data review committee will grant access after the review and the data use agreement is finalized.

## Supplementary Materials

The following supporting information can be downloaded at: https://www.mdpi.com/article/10.3390/ijms27031574/s1.
