# Supplementary material for: Genetic Insights into Circulating Complement Proteins in Myalgic Encephalomyelitis/Chronic Fatigue Syndrome: A Potential Inflammatory Subgroup
Source: Int J Mol Sci. 2026 Feb 5;27(3):1574. doi: 10.3390/ijms27031574 (PMC12898610; doi:10.3390/ijms27031574)
Supplement: Supplementary file 1 [file ijms-27-01574-s001.zip › Supplementary_Figures_IJMS_Complement.pdf]

Supplementary Materials

Genetic Insights into Circulating Complement Proteins in Myalgic Encephalomyelitis/Chronic Fatigue Syndrome: A potential inflammatory subgroup

Jessica Maya[1,*], Elizabeth R. Unger[1], Jin-Mann S. Lin[1], and Mangalathu S. Rajeevan[1,*]

[1] Division of High-Consequence Pathogens & Pathology, Centers for Disease Control & Prevention, Atlanta, GA 30329, USA; dwe3@cdc.gov

* Correspondence: xpa0@cdc.gov, msrajeevan53@gmail.com

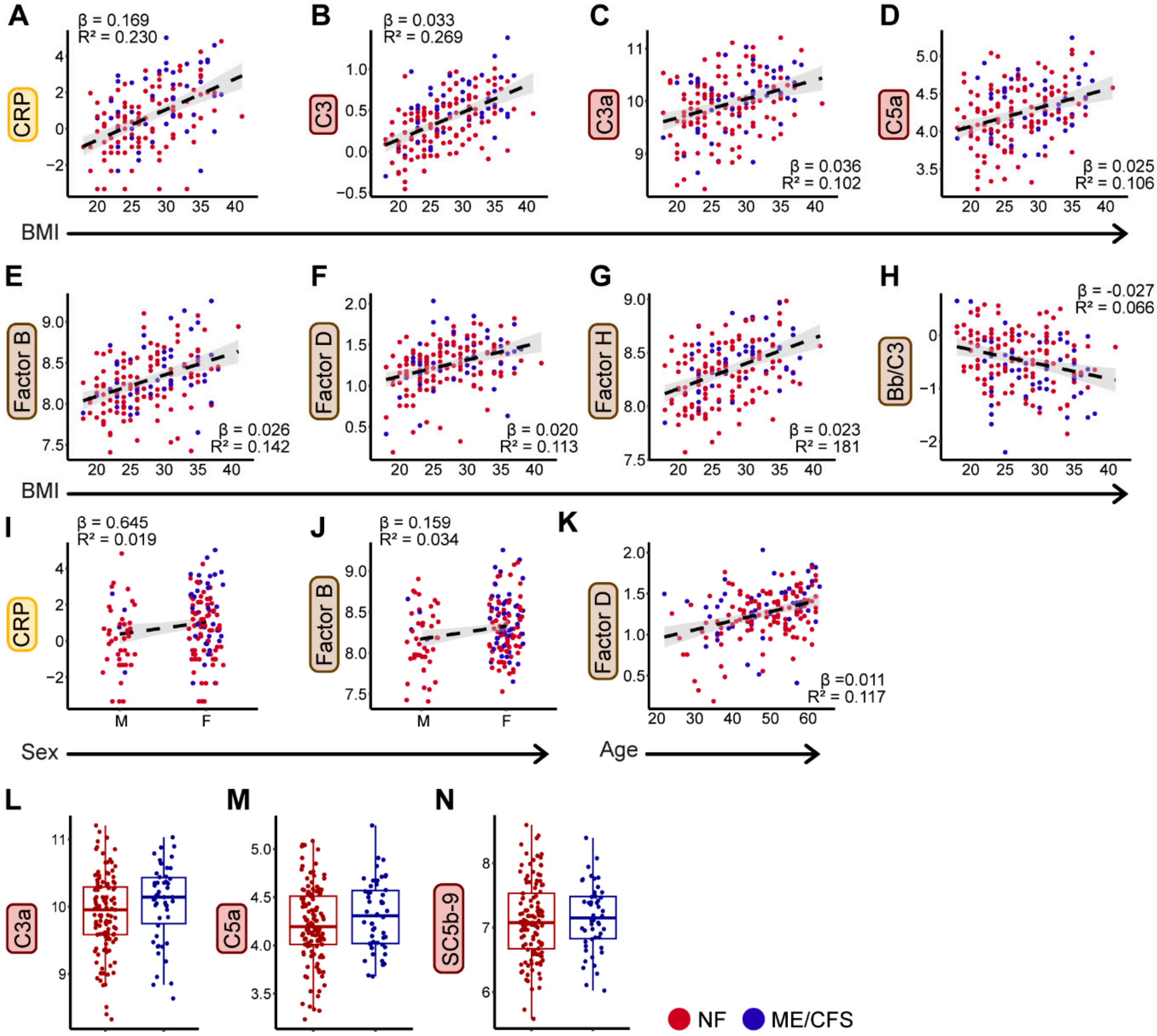

Figure S1. Identification of covariates significantly associated with circulating complement protein levels in all subjects. Dot plots depicting covariate associations between BMI and log2-transformed plasma levels of **(A)** CRP (mg/L), **(B)** C3

(mg/ml), **(C)** C3a (ng/ml), **(D)** C5a (ng/ml), **(E)** Factor B (ug/ml), **(F)** Factor D (ug/ml), **(G)** Factor H (ug/ml), and **(H)** Bb/C3. Dot plots depicting covariate associations between sex and log2-transformed plasma levels of **(I)** CRP (mg/L) and **(J)** Factor B (ug/ml). **(K)** Dot plot depicting covariate associations between age and log2-transformed plasma levels of Factor D(ug/ml). **(L)** C3a levels in plasma (p_{lin}=0.38, p_{log}=0.37, log2, ng/ml) compared between NF and ME/CFS. **(M)** C5a levels in plasma (p_{lin}=0.48, p_{log}=0.49, log2, ng/ml) compared between NF and ME/CFS. **(N)** SC5b-9 complex levels in plasma (p_{lin}=0.69, p_{log}=0.69, log2, ng/ml) compared between NF and ME/CFS. Each dot represents an individual (NF = red, ME/CFS = blue). Statistical comparisons of circulating complement protein levels with covariates were performed using linear regression models, with regression lines and 95% confidence intervals overlaid. Regression outputs (including p-values) are listed in Table 1. Proteins significantly associated with BMI, sex, and age were adjusted for in all subsequent analyses. Boxplots represent the median ± 25th and 75th quartiles. Whiskers represent 1.5x the interquartile ranges. Outliers are values outside the whisker range. Statistical comparisons of circulating complement protein levels were performed using co-variate-adjusted (Table 1) linear (p_{lin}) and logistic (p_{log}) regression analysis.

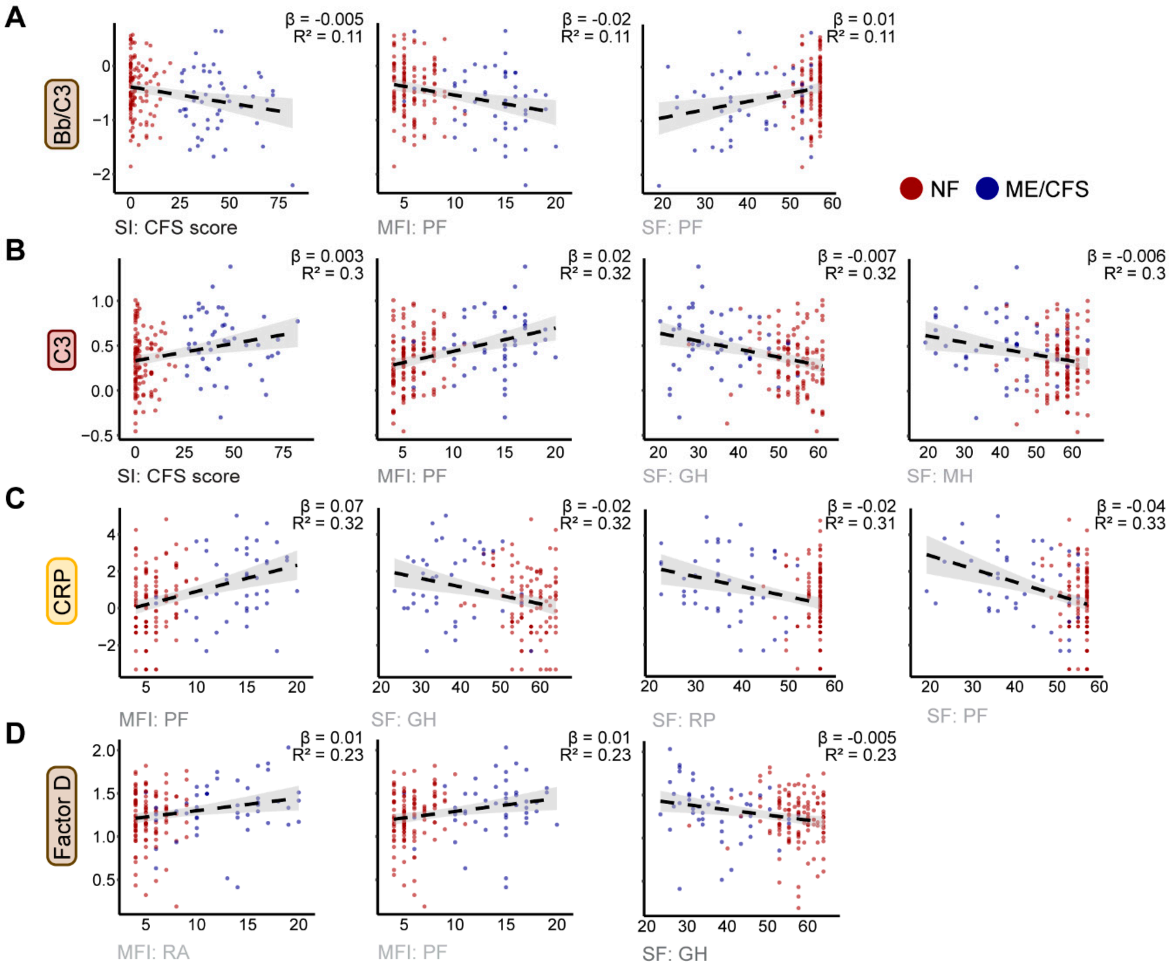

Figure S2. Circulating complement protein levels are significantly associated with scores of symptoms, function, and fatigue. **(A)** Dot plots of plasma Bb/C3 levels (log2) compared to: (left to right) Symptom Inventory (SI): CFS score, MFI: PF, and SF: PF scores. Each dot represents an individual (NF = red, ME/CFS = blue). Linear regression lines with 95% confidence intervals are overlaid. Y-axis font colors are coded to Figure 2 labels for instruments. **(B)** Dot plots of plasma C3 levels (log2, mg/ml) compared to: (left to right) SI: CFS score, MFI: PF, SF: GH, and SF: MH scores. **(C)** Dot plots of plasma CRP levels (log2, mg/L) compared to: (left to right) MFI: PF, SF: GH, SF: RP, and SF: PF scores. **(D)** Dot plots of plasma Factor D levels (log2, ug/ml) compared to: (left to right) MFI: RA, MFI: PF, and SF: GH scores. (SI metrics: CFS score = sum of CFS-defining symptom scores; MFI metrics: MF = mental fatigue, RM = reduced motivation, RA = reduced activity, PF = physical fatigue, GF = general fatigue; SF-36 metrics: MH = mental health, SF = social functioning, RE = role: emotional, GH = general health, V = vitality, BP = bodily pain, RP = role: physical, PF = physical functioning) NOTE: Complement protein labels are color coded to match components of complement system illustrated in Figure 1E.

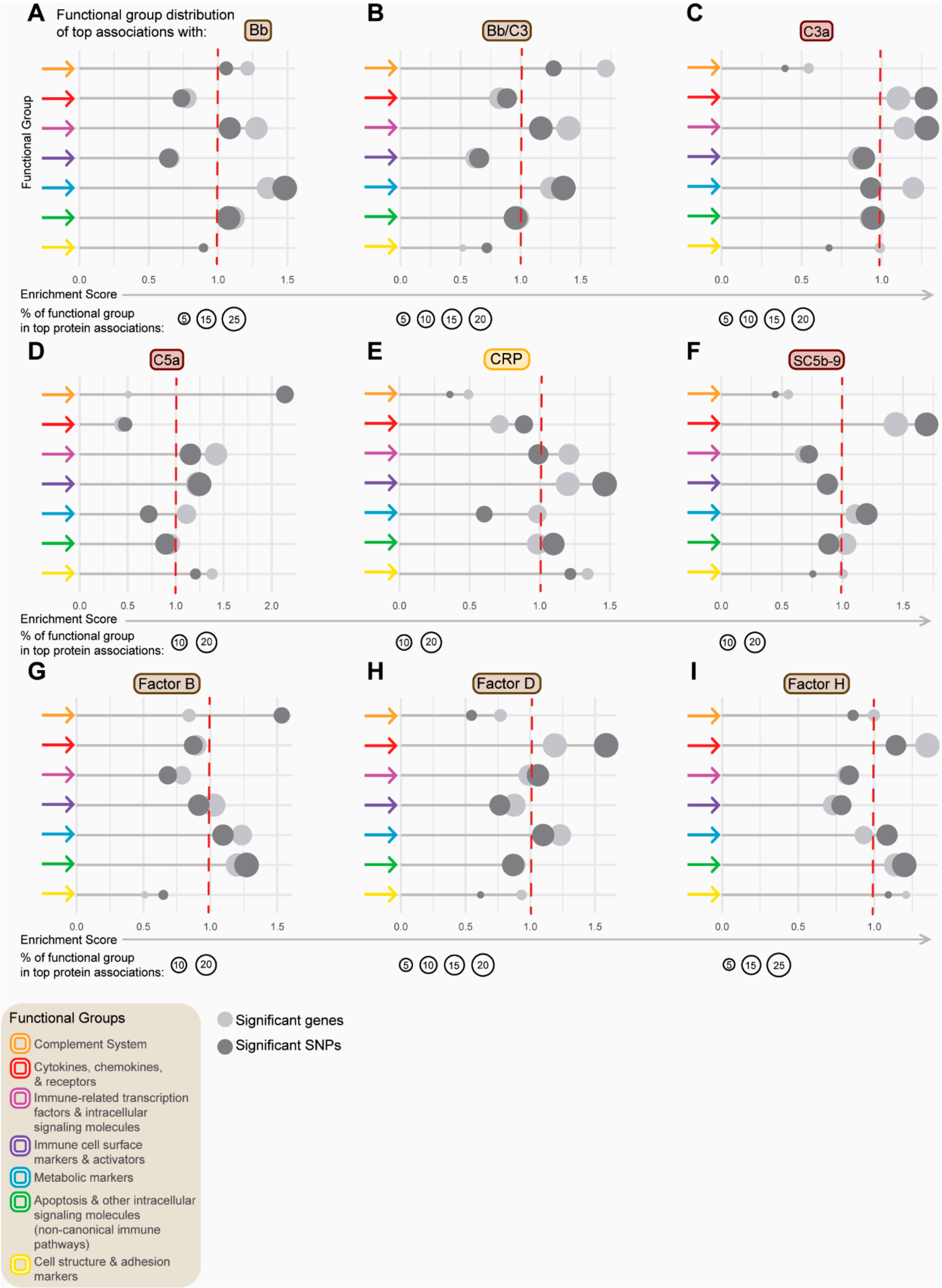

A
Functional group distribution of top associations with:
Bb
B
Bb/C3
C
C3a
D
C5a
E
CRP
F
SC5b-9
G
Factor B
H
Factor D
I
Factor H
Functional Group
Enrichment Score
% of functional group in top protein associations:
0.0
0.5
1.0
1.5
2.0
Functional Groups
Complement System
Cytokines, chemokines, & receptors
Immune-related transcription factors & intracellular signaling molecules
Immune cell surface markers & activators
Metabolic markers
Apoptosis & other intracellular signaling molecules (non-canonical immune pathways)
Cell structure & adhesion markers
Significant genes
Significant SNPs

Figure S3. Functional group enrichment of genetic variants significantly associated with circulating complement protein levels. Lollipop plot showing functional group enrichment among top SNPs associated with **(A)** Bb, **(B)** Bb/C3, **(C)** C3a, **(D)** C5a, **(E)** CRP, **(F)** SC5b9, **(G)** Factor B, **(H)** Factor D, and **(I)** Factor H plasma levels. Dot size reflects the proportion of significant genes (light green) and SNPs (dark green) in in total significant protein-associated SNPs for the linked functional group. Enrichment scores on the x-axis compare observed hits within each group for specific complement protein-associated SNPs ($p \leq 0.01$) to expected counts across all groups, with overrepresentation in a group indicated by an enrichment score > 1 (dotted red line). See Tables S1-11.

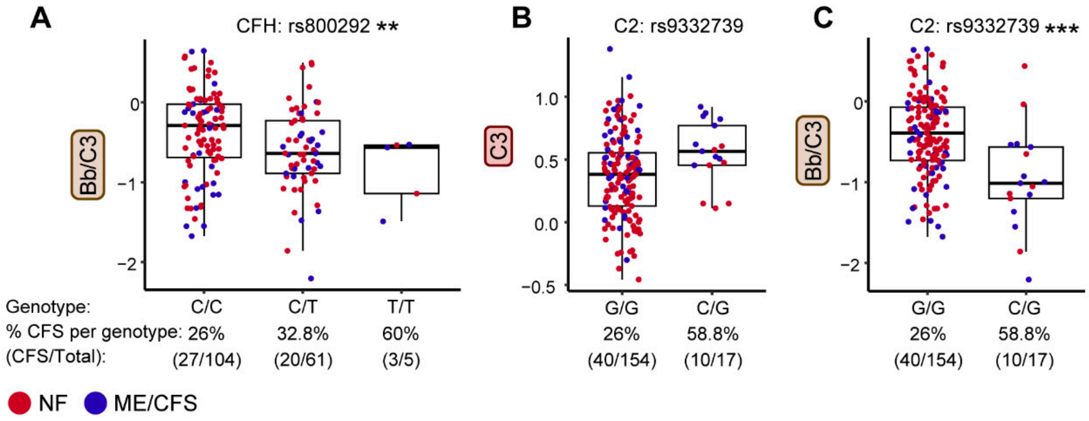

Figure S4. Genetic variants associated with ME/CFS and circulating complement protein levels. **(A)** Boxplot showing genotype-dependent differences in the ratio of plasma Bb levels to C3 levels for rs800292 in CFH (*p*=0.003, log2), a top SNP from the complement system group for Bb/C3 associations. The percentage of ME/CFS subjects within each genotype group is indicated on the x-axis (NF = red, ME/CFS = blue) while the CFS/Total row indicates the number of ME/CFS subjects vs the total number of subjects per genotype. **(B)** Boxplots showing genotype-dependent differences in plasma C3 levels for rs9332739 in C2 (*p*=0.11, log2, mg/ml). **(C)** Boxplots showing genotype-dependent differences in the ratio of plasma Bb levels to C3 levels for rs9332739 in C2 (*p*=0.0002, log2). Boxplots represent the median ± 25th and 75th quartiles. Whiskers represent 1.5x the interquartile ranges. Outliers are values out-side the whisker range. Asterisks indicate a significant overall genotype effect based on a covariate-adjusted full-versus-reduced (FvR) linear regression model comparing models with and without the genotype term. Significance (p-value) reflects improvement in model fit attributable to genotype and does not represent pairwise comparisons between individual genotype groups. *p≤0.05, **p≤0.01, ***p ≤0.001.

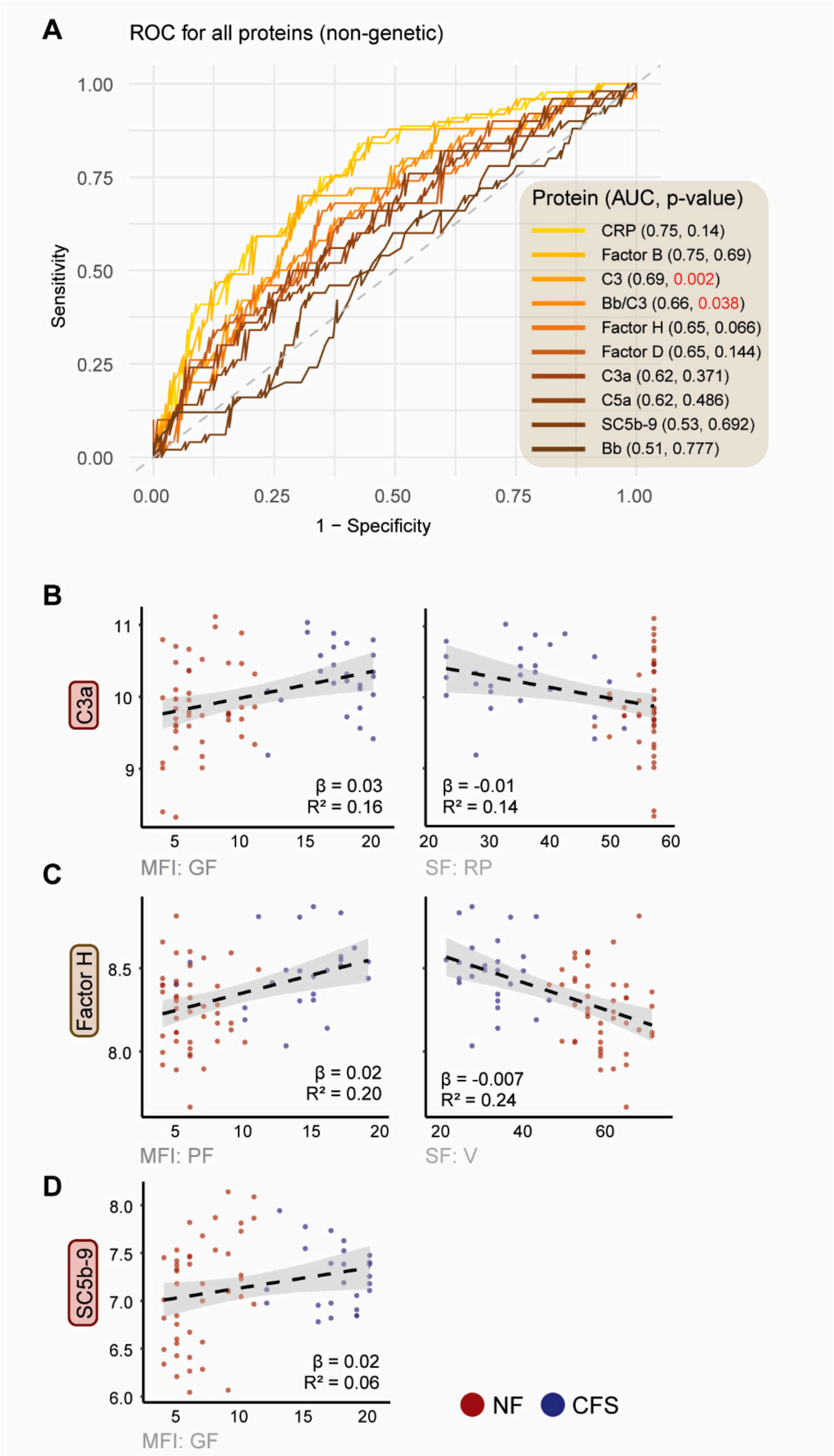

Figure S5. Predictive power of circulating complement proteins on disease and functional health score associations with complement proteins in genotype-stratified subgroups. **(A)** ROC curve analysis comparing the predictive accuracy of each circulating complement protein in distinguishing ME/CFS from control participants, without

incorporating genotype information (n = 171 total; 50 ME/CFS, 121 NF). Sensitivity and specificity values are plotted for each protein, with AUC values and p-values displayed in the legend (red = significant). **(B)** Dot plots of plasma C3a levels (log2, ng/ml) compared to: (left to right) MFI: GF and SF: RP scores. Each dot represents an individual (NFhet = red, CFShet = blue). Linear regression lines with 95% confidence intervals are overlaid. Y-axis font colors are coded to Figure 5 labels for instruments **(C)** Dot plots of plasma Factor H levels (log2, ug/ml) compared to: (left to right) MFI: PF and SF: V scores. **(D)** Dot plot of plasma SC5b9 levels (log2, ng/ml) compared to MFI: GF score. (SI metrics: CFS score = sum of CFS-defining symptom scores; MFI metrics: MF = mental fatigue, RM = reduced motivation, RA = reduced activity, PF = physical fatigue, GF = general fatigue; SF-36 metrics: MH = mental health, SF = social functioning, RE = role: emotional, GH = general health, V = vitality, BP = bodily pain, RP = role: physical, PF = physical functioning)

Supplementary tables are included via Microsoft Excel file: Supplementary_Tables_IJMS_Complement.xlsx

Table S1. Pearson correlation analysis of all circulating plasma protein levels (inter-protein analysis) in total study samples. Red text indicates significant p-value below 0.05. Green text indicates the inclusion of this correlation (plotted) in the main text (Figure 1L).

Table S2: Top SNP associations with plasma Bb levels (linear regression, p>0.05, adjusted for covariates)

Table S3. Top SNP associations with plasma Bb/C3 levels (linear regression, p>0.05, adjusted for covariates)

Table S4. Top SNP associations with plasma C3 levels (linear regression, p>0.05, adjusted for covariates)

Table S5. Top SNP associations with plasma C3a levels (linear regression, p>0.05, adjusted for covariates)

Table S6. Top SNP associations with plasma C5a levels (linear regression, p>0.05, adjusted for covariates)

Table S7. Top SNP associations with plasma CRP levels (linear regression, p>0.05, adjusted for covariates)

Table S8. Top SNP associations with plasma Factor B levels (linear regression, p>0.05, adjusted for covariates)

Table S9. Top SNP associations with plasma Factor D levels (linear regression, p>0.05, adjusted for covariates)

Table S10. Top SNP associations with plasma Factor H levels (linear regression, p>0.05, adjusted for covariates)

Table S11. Top SNP associations with plasma SC5b-9 levels (linear regression, p>0.05, adjusted for covariates)

Table S12. Gene annotations and category assignments used for functional group enrichment plots.

Table S13. List of 51 SNPs significantly associated with disease (reference 3) used for Fig.4B-D
